# Evaluation of AlphaFold antibody–antigen modeling with implications for improving predictive accuracy

**DOI:** 10.1002/pro.4865

**Published:** 2024-01-01

**Authors:** Rui Yin, Brian G. Pierce

**Affiliations:** ^1^ University of Maryland Institute for Bioscience and Biotechnology Research Rockville Maryland USA; ^2^ Department of Cell Biology and Molecular Genetics University of Maryland College Park Maryland USA

**Keywords:** antibody, antigen, AlphaFold, deep learning

## Abstract

High resolution antibody–antigen structures provide critical insights into immune recognition and can inform therapeutic design. The challenges of experimental structural determination and the diversity of the immune repertoire underscore the necessity of accurate computational tools for modeling antibody–antigen complexes. Initial benchmarking showed that despite overall success in modeling protein–protein complexes, AlphaFold and AlphaFold‐Multimer have limited success in modeling antibody–antigen interactions. In this study, we performed a thorough analysis of AlphaFold's antibody–antigen modeling performance on 427 nonredundant antibody–antigen complex structures, identifying useful confidence metrics for predicting model quality, and features of complexes associated with improved modeling success. Notably, we found that the latest version of AlphaFold improves near‐native modeling success to over 30%, versus approximately 20% for a previous version, while increased AlphaFold sampling gives approximately 50% success. With this improved success, AlphaFold can generate accurate antibody–antigen models in many cases, while additional training or other optimization may further improve performance.

## 1 INTRODUCTION

Antibodies are a key component of the immune system, defending the host from viruses and other pathogens through specific recognition of protein and non‐protein antigens. Typically, antibodies engage their antigenic targets using the hypervariable complementarity determining region (CDR) loops within the variable domain (Chothia & Lesk, 1987), which are stabilized by the β‐sandwich structure of the framework region (Sela‐Culang et al., 2013). Despite sharing a conserved immunoglobulin structure, antibodies collectively exhibit a remarkable ability to recognize and bind to a wide array of antigens with high specificity. The highly specific and diverse nature of antibody–antigen interactions makes antibodies highly useful as therapeutics as well as a consideration in vaccine development efforts (Carter, 2006; Nelson et al., 2010; Rappuoli et al., 2016; Scott et al., 2012).

High resolution structures of antibody–antigen complexes have refined our knowledge of immunity (Li et al., 2003), revealed molecular basis of antibody recognition of viral epitopes (Barnes et al., 2020; Dreyfus et al., 2012; Zhou et al., 2015), and guided the effective design of antibodies (Haidar et al., 2012; Hanf et al., 2014) and immunogens (Graham et al., 2019). However, due to the challenges of experimental structure determination, resource and time constraints, as well as the highly diverse nature of the immune repertoire (Georgiou et al., 2014; Li et al., 2004), experimental characterization of most antibody–antigen complex structures is impractical. Therefore, computational tools have been developed and applied to bridge this gap. General protein–protein docking methods have been applied to model antibody–antigen complex structures with limited success (Vreven et al., 2015), due in part to the need to account for the mobility of key CDR loops, as well as the size of certain antigens. To address this, algorithms have been developed specifically for antibody–antigen complex modeling (Ambrosetti et al., 2020; Brenke et al., 2012; Krawczyk et al., 2013; Sircar & Gray, 2010). However, accurate structural prediction of antibody–antigen complexes remains a challenge (Guest et al., 2021; Vreven et al., 2015).

Recently, the scientific community saw a major breakthrough with AlphaFold (v.2.0), which uses an end‐to‐end deep neural network to predict protein structures from sequence (Jumper et al., 2021a). AlphaFold iteratively infers and refines pairwise residue–residue evolutionary and geometric information from multiple sequence alignments (MSAs) and has achieved unprecedented success in protein structure prediction (Jumper et al., 2021a, 2021b). Its capabilities were expanded by the development of AlphaFold‐Multimer (Evans et al., 2021) (released in AlphaFold v.2.1), an updated implementation of AlphaFold that was designed to predict protein–protein complex structures. The overall architecture of AlphaFold‐Multimer is similar to the previous version of AlphaFold, with changes including cross‐chain MSA pairing, adjusted loss functions, and training on protein–protein interface residues.

Previously, our benchmarking revealed that, while generally successful in protein–protein complex structure prediction, AlphaFold was less successful in modeling antibody–antigen complexes, and adaptive immune recognition in general (Yin et al., 2022). This lack of success in antibody–antigen structure prediction was also noted by the developers of AlphaFold‐Multimer (Evans et al., 2021). However, some highly accurate antibody–antigen complex models were generated by AlphaFold (Yin et al., 2022), which shows potential for success of the “fold‐and‐dock” approach for antibody–antigen structure prediction. While recent studies have assessed the predictive performance of AlphaFold for modeling unbound antibodies (Abanades et al., 2023; Ruffolo et al., 2023), or optimization of AlphaFold's ability to predict protein complexes in general (Bryant et al., 2022; Wallner, 2023a), studies have not focused on AlphaFold's performance in antibody–antigen recognition, particularly in light of updated versions of AlphaFold and its multimer model (v2.2, v2.3) (DeepMind, 2022), since our initial test of AlphaFold v2.1 on a set of 100 antibody–antigen complexes (Yin et al., 2022). Thus, there is a need for an updated and expanded benchmarking and analysis of AlphaFold performance on this challenging and important class of complexes.

In this study, we report a comprehensive benchmarking of AlphaFold for antibody–antigen complex structure modeling. With a dataset of over 400 high resolution and non‐redundant antibody–antigen complexes, representing a major increase over the 100 complexes that were used previously (Yin et al., 2022), we investigated factors contributing to modeling successes and failures, including antibody class and subunit accuracy. The default AlphaFold model confidence score was found to be well correlated with antibody–antigen model accuracy, while residue‐level confidence for interface residues was likewise correlated with model accuracy. Interestingly, we found that recent optimization of AlphaFold led to notably higher antibody–antigen accuracy, while use of a “massive sampling” strategy with large sets of pooled AlphaFold models for each complex (Wallner, 2023a) led to even better performance. Our study presents a thorough analysis of AlphaFold's ability to predict antibody–antigen complexes, yielding valuable insights for interpreting model accuracy, identifying obstacles in the modeling process, and highlighting potential areas for improvement.

## 2 RESULTS

### 2.1 AlphaFold antibody–antigen complex modeling accuracy

To perform a comprehensive and detailed assessment of AlphaFold's ability to model antibody–antigen complexes, we assembled a set of over 400 nonredundant antibody–antigen complexes released after April 30, 2018 (Table S1). The date cutoff was selected to avoid overlap with the training set of the tested version of AlphaFold (v2.2.0, hereafter denoted as v2.2 for brevity). Nonredundancy and additional test case selection criteria are described in Section 4. For efficiency, we only utilized the variable domains of the antibody sequences for modeling. As all AlphaFold modeling of multimers in this study was performed with the multimer model of AlphaFold, we use the term AlphaFold (vs. AlphaFold‐Multimer) to denote that protocol in this study, for brevity. The accuracy of antibody–antigen complex predictions was evaluated using Critical Assessment of Predicted Interactions (CAPRI) criteria (Lensink et al., 2020), which classify predictions as incorrect, acceptable, medium, or high based on a combination of interface root‐mean‐square distance (I‐RMSD), ligand root‐mean‐square distance (L‐RMSD), and fraction of native interface residue contacts (fnat), in comparison with the experimentally determined antibody–antigen complex structure.

**FIGURE 1:**
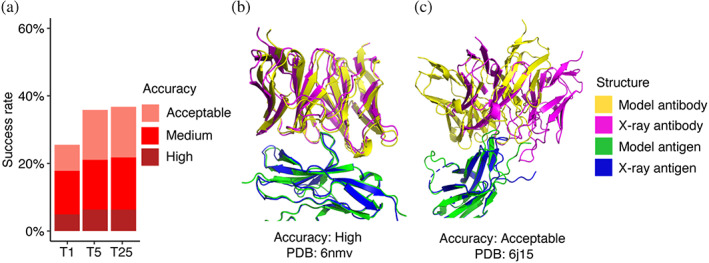
Antibody–antigen modeling accuracy of AlphaFold. (a) Benchmarking of AlphaFold (v.2.2, multimer model) was performed on 427 antibody–antigen complexes. For each complex, 25 predictions were generated and ranked by AlphaFold model confidence score. Antibody–antigen predictions were evaluated for complex modeling accuracy using CAPRI criteria for high, medium, and acceptable accuracy. The success rate was calculated based on the percentage of cases that had at least one model among their top *N* ranked predictions that met a specified level of CAPRI accuracy. Bars are colored by CAPRI accuracy level. (b) Example of a near‐native prediction by AlphaFold, in comparison with the experimentally determined structure (PDB: 6nmv; antibody/SIRP‐alpha complex). This model has high CAPRI accuracy (I‐RMSD = 0.68 Å) and has the highest model confidence of all 25 predictions of this complex (model confidence = 0.88). (c) An example of an acceptable accuracy complex model from AlphaFold, in comparison with the experimentally determined structure (PDB: 6j15; antibody/PD‐1 complex). This model has acceptable CAPRI accuracy (I‐RMSD = 3.35 Å), and has the highest model confidence of all 25 predictions of this complex (model confidence = 0.75). Complex structures in (b,c) are superposed by antigen with the model and the x‐ray structure components colored separately as indicated on right.

AlphaFold generated acceptable or higher accuracy models as top‐ranked predictions for 26% of the 427 test cases for which models were generated (Figure 1a). Medium or higher accuracy models, which we refer to as near‐native predictions, were generated as top‐ranked predictions for 18% of the cases, and high accuracy models were generated for 5% of the test cases. Success rates increased when all 25 predictions per complex were taken into consideration, leading to 37% of the cases achieving acceptable or higher accuracy predictions, 22% achieving medium or higher accuracy predictions, and 6% achieving high accuracy predictions.

Representative models generated by AlphaFold are shown in Figure 1b (PDB code 6nmv; antibody/SIRP‐alpha complex) and Figure 1c (PDB code 6j15; antibody/PD‐1 complex). Both models are top‐ranked predictions for the respective complex. The model in Figure 1b has high CAPRI accuracy, and an interface root‐mean squared distance (I‐RMSD) value of 0.68 Å, indicating a low level of structural deviation of this modeled antibody–antigen complex from the native complex. Figure 1c shows an acceptable CAPRI accuracy prediction with an I‐RMSD of 3.55 Å. While the antibody engages the correct site of the antigen in this example, a deviation in positioning of the antibody on the antigen, with respect to the experimentally determined structure, is observed.

We compared the AlphaFold benchmarking results with other pipelines and approaches, including AlphaFold in ColabFold (Mirdita et al., 2022). For fairness of the comparison with the full AlphaFold pipeline's results, ColabFold was modified to generate 25 predictions per complex. ColabFold's modeling success was similar to that of AlphaFold for 426 cases for which both algorithms were able to generate models, with slightly lower success observed for ColabFold (Figure S1). The difference in success may be due to factors such as different MSAs or structural templates, as ColabFold and AlphaFold employ distinct approaches for building and pairing MSAs, and utilize different sequence and template databases. In a comparison with previously developed docking approaches, we observed that AlphaFold exhibits higher success in antibody–antigen modeling than rigid‐body docking algorithms ZDOCK (Pierce et al., 2011) and ClusPro (antibody mode) (Brenke et al., 2012) with modeled unbound structures as input (Supplementary Results, Figures S2 and S3).

We observed higher success in modeling antibody–antigen complexes by AlphaFold in this study versus our previous benchmarking study, in which fewer than 10% of cases had top‐ranked predictions with near‐native accuracy (Yin et al., 2022) (vs. 18% here, as noted above). This difference is likely due to the newer version of AlphaFold used in this study (v2.2 vs. v2.1), which uses a retrained multimer model, as well as different sets of test cases, with the current study representing a substantial expansion over the cases used previously.

### 2.2 Antibody–antigen modeling accuracy determinants

**FIGURE 2:**
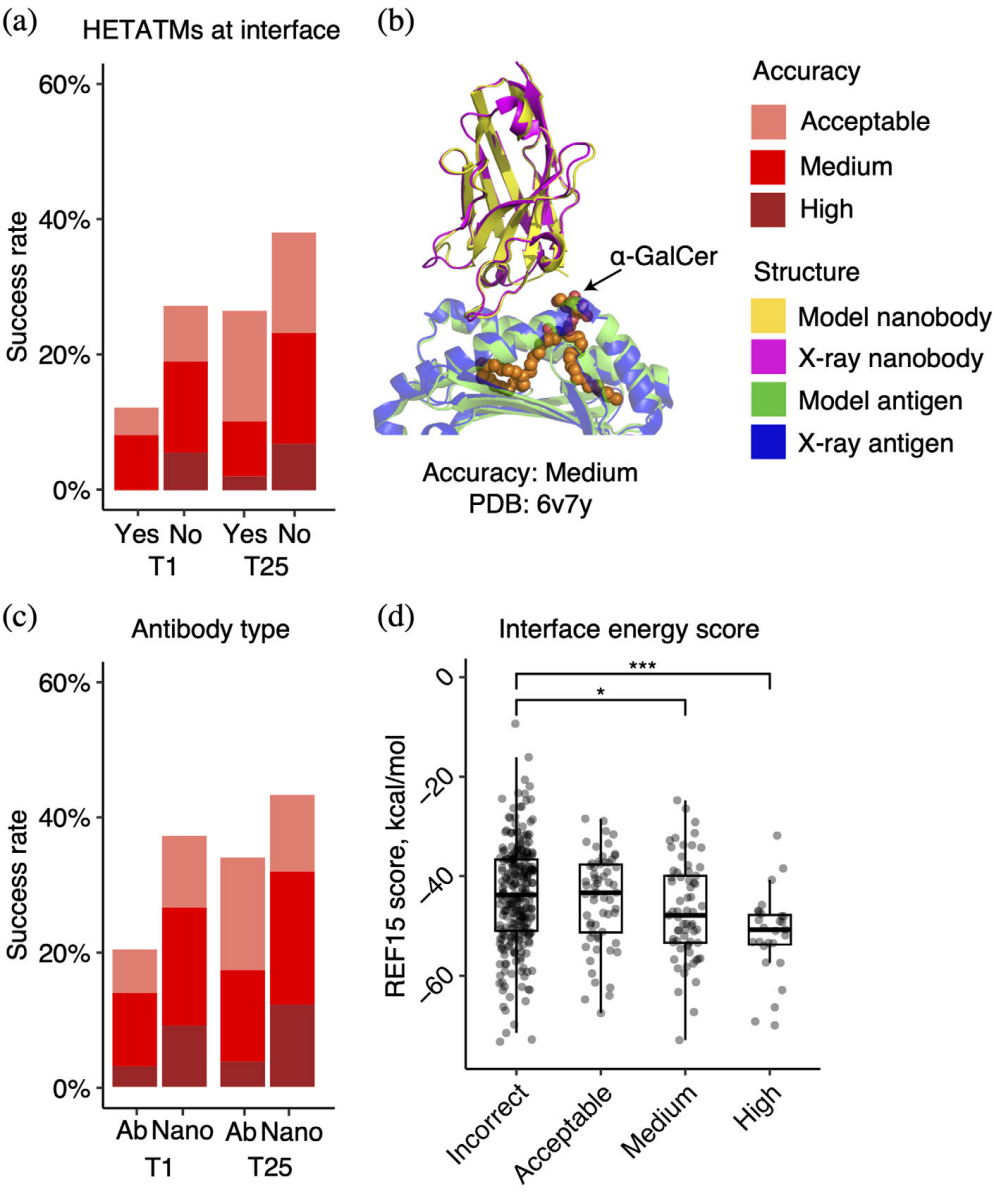
Properties associated with antibody–antigen modeling success. (a) Success rates based on presence of non‐protein atoms (glycans or ligands) at the antibody–antigen interface. Complexes are classified as either “Yes” (*N* = 49) or “No” (*N* = 378) to indicate whether glycans/ligands are present or absent in the antibody–antigen interface. (b) An example of a medium accuracy complex model from AlphaFold for an interface ligand complex, in comparison with the experimentally determined structure (PDB: 6v7y; single‐domain antibody/CD1d α‐GalCer complex). This model has medium CAPRI accuracy (I‐RMSD = 1.02 Å), and has the highest model confidence of all 25 predictions of this complex (model confidence = 0.85). The complex structure is superposed by antigen with the model and the x‐ray structure components colored separately as indicated on right. The α‐GalCer glycolipid from the x‐ray structure is colored orange. (c) Success rates based on type of antibody in the complex. Complexes were classified as “Ab” (heavy‐light antibody, *N* = 295), or “Nano” (nanobody/VHH, *N* = 132) based on antibody type. T1 and T25 denote AlphaFold modeling accuracy in top 1 (ranked by AlphaFold model confidence score) and in all 25 predictions of the complex. Bars were colored by CAPRI criteria. (d) Distribution of Interface energy score calculated by the Rosetta InterfaceAnalyzer (Stranges & Kuhlman, 2013) protocol (based on Rosetta REF15 energy function [Alford et al., 2017]) grouped by AlphaFold modeling accuracy. The modeling accuracy is defined as the highest CAPRI criteria prediction in the complex, considering all 25 predictions. Statistical significance values (Wilcoxon rank‐sum test) were calculated between interface energy scores for sets of cases with incorrect versus medium and incorrect versus high CAPRI accuracy predictions, as noted at top (*p ≤ 0.05; ***p ≤ 0.001).

To identify possible factors associated with modeling outcome, we analyzed properties of the native antibody–antigen complexes in relation to predictive modeling success. As glycans are not modeled by AlphaFold and antigen glycosylation can be an important component in antibody–antigen recognition (in some cases with glycans contacted directly by antibodies) (Kappler & Hennet, 2020), the subset of complexes with antibody–antigen interface glycans in our set was identified (*N* = 45) to assess the impact of antigen glycosylation on modeling outcome. We identified several additional cases (*N* = 4) containing non‐protein ligand molecules (lipids, nucleotides) at the antibody–antigen interface that were likewise included in the set. Our analysis showed that the presence of non‐protein ligands and glycans at the native antibody–antigen interface is associated with lower modeling success (Figure 2a). Among those 49 cases, the top‐ranked predictions of medium accuracy were produced in only 8% of the cases, and no high accuracy top‐ranked predictions were produced. In contrast, for cases not belonging to this category, 19% had top‐ranked predictions of medium or higher accuracy. Thus, the lack of explicit consideration of interface glycans and ligands may reduce modeling accuracy for some antibody–antigen complexes. Nonetheless, AlphaFold was able to accurately model a single‐domain antibody–antigen complex for which the native structure contains with a glycosphingolipid antigen α‐galactosylceramide (α‐GalCer) in the binding interface, as shown in Figure 2b (PDB code 6v7y; single‐domain antibody/CD1d α‐GalCer complex). The model, a top‐ranked prediction for the complex, has medium CAPRI accuracy, and an I‐RMSD value of 1.02 Å, indicating that AlphaFold accurately captured the antibody–antigen docking conformation despite the absence of an explicit representation of the glycosphingolipid antigen at the binding interface.

Antigen glycosylation can be an important component in antibody–antigen recognition, with many cases of glycans contacted directly by antibodies (Kappler & Hennet, 2020). The importance of glycans, as well as the prevalence of antigen N‐glycosylation in our dataset (45 out of 49 glycan/ligand interface complexes, as noted above), prompted us to examine antigen glycosylation in the set further. As some x‐ray or cryo‐EM structures used for analysis may lack resolved glycan atoms, or naturally occurring glycans can be removed enzymatically or via mutation to enable structural characterization, it is possible that some members of the non‐glycan/ligand set (*N* = 378) may actually have interface‐proximal glycans in vivo. Based on the analysis of antigen source organism and proximity of surface‐exposed *N*‐glycosylation motifs to the interacting antibody, a subset of *N* = 91 cases were identified to have possible antigen *N*‐glycosylation near antibody‐binding site. The predicted antibody‐proximal antigen glycosylation subset showed a moderately lower modeling success, with medium or higher accuracy top‐ranked predictions generated in 16% of cases, compared to 20% medium/high success for the cases without likely or structurally resolved interface *N*‐glycosylation (*N* = 287) (Figure S4). It should be noted that factors such as varying levels of *N*‐glycan site occupancy and cellular localization (e.g. intracellular vs. extracellular proteins) were not considered in the computational identification of potential *N*‐glycan sites, and experimental methods such as mass spectroscopy of native proteins from organism‐specific cells would be needed for more conclusive identification of antigen‐linked glycans.

We also investigated whether antibody–antigen complexes containing single‐chain antibodies (or nanobodies) are more successfully modeled compared to the heavy–light chain only counterparts (Figure 2c). For nanobody–antigen complexes (*N* = 132), 27% of cases had medium or higher accuracy top‐ranked predictions, versus 14% of cases with medium or higher accuracy top‐ranked predictions for heavy‐light chain antibody–antigen complexes (*N* = 295). To understand the pronounced difference in modeling nanobody–antigen complexes versus antibody–antigen complexes, we investigated the difference in MSA depth of the two types of complexes. We hypothesized that the single‐chain variable domains in nanobodies may simplify construction of cross‐chain MSAs for nanobody–antigen complexes, as opposed to the more complex heavy–light chain antibodies. However, after analyzing the MSA depth, we found no statistically significant difference in the number of effective sequences (N_eff_, a measure of the effective sequence count in an MSA [Jumper et al., 2021a]) between the two types of complexes (Figure S5). This suggests that other factors, such as fewer CDR loops and a smaller search space, may contribute to the observed difference in modeling success. Unlike heavy–light chain antibodies, which possess six CDR loops, the variable domain of nanobodies contains three loops only, thus it is possible that the lower complexity and size of the receptor component of the complex may play a role in the observed improved modeling performance for AlphaFold.

To investigate whether more favorable antibody–antigen interfaces are more successfully predicted by AlphaFold, we compared antibody–antigen interface energy, computed from the bound complex structure using Rosetta (Leman et al., 2020), with modeling success considering all 25 predictions of each case (Figure 2d). We found that more negative interface energies, indicative of more energetically favorable protein–protein interactions, are associated with higher AlphaFold modeling success. The difference in distribution of interface energy scores between complexes is statistically significant between incorrect versus medium accuracy prediction (*p* ≤ 0.05), and incorrect versus high accuracy complexes (*p* ≤ 0.001), based on Wilcoxon rank‐sum test. Other factors including modeled antigen assembly mode, CDR loop modeling accuracy, and antigen modeling accuracy were evaluated for their influence on the success of antibody–antigen modeling (Supplementary Results, Figures S6–S8).

### 2.3 Model confidence score comparison

**FIGURE 3:**
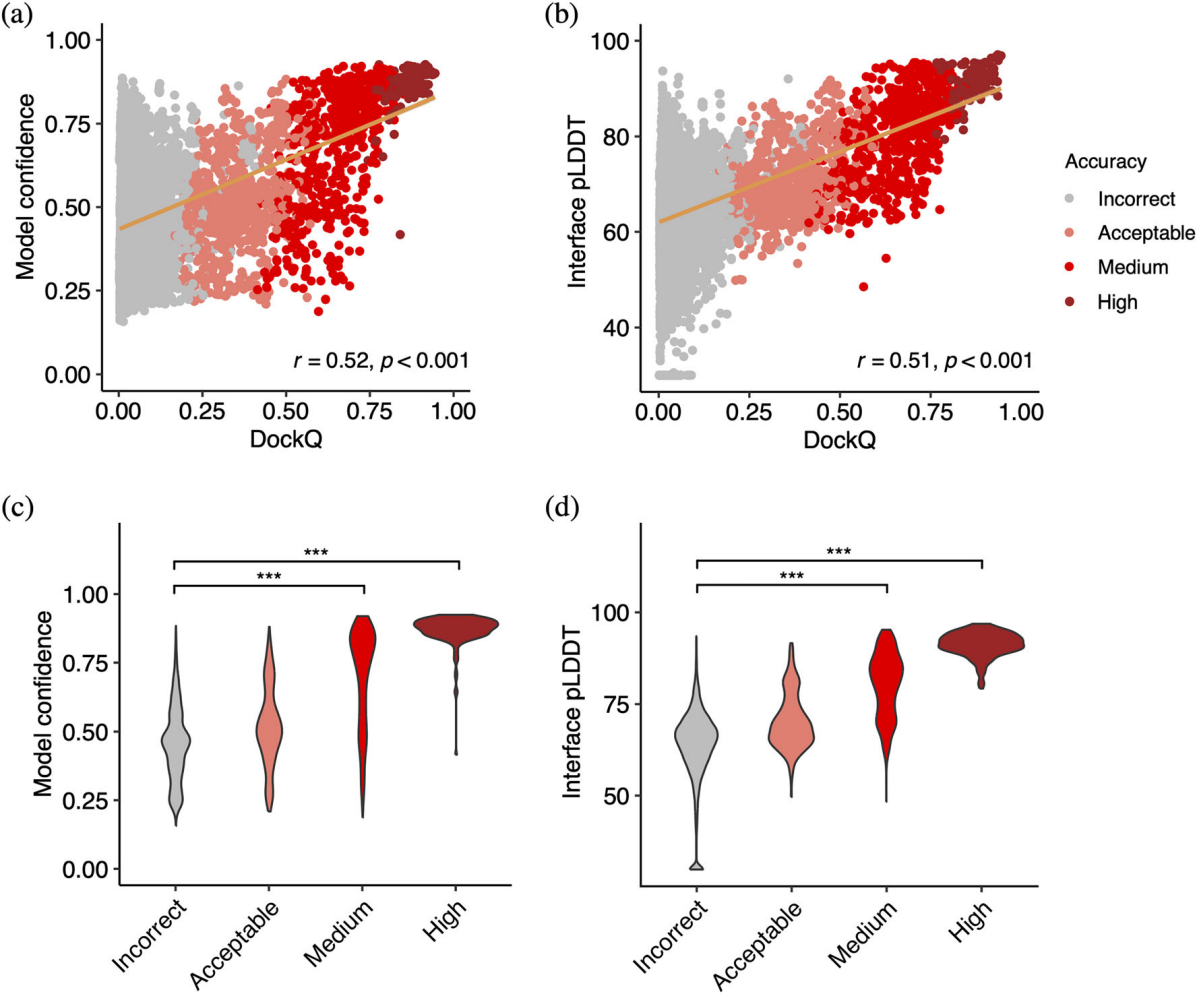
AlphaFold model confidence scores and model accuracy. Scatter plots compare (a) model confidence and (b) interface pLDDT score with model accuracy, with accuracy assessed by DockQ score. In the scatter plots, all 25 models representing 427 complexes are depicted as data points, with their colors indicating the model quality according to CAPRI criteria. The orange line represents the linear regression, and the lower right corner of the scatter plots displays the Pearson's correlation coefficients and correlation *p*‐values. Distribution of (c) model confidence and (d) interface pLDDT score, grouped by the CAPRI criteria of AlphaFold predictions. Interface pLDDT score is defined as the mean of pLDDT scores of residues within 4 Å of the antibody–antigen interface. Complexes without contacts within 4 Å of antibody–antigen interface is assigned an I‐pLDDT score of 30. Statistical significance values (Wilcoxon rank‐sum test) were calculated between model scores for sets of predictions with incorrect versus medium and incorrect versus high CAPRI accuracy, as noted at top (***p ≤ 0.001).

The reported success of model accuracy scores produced by AlphaFold (Evans et al., 2021; Yin et al., 2022) led us to evaluate the ability of those scores, or adaptations thereof, to discriminate between accurate versus incorrect antibody–antigen predictions. We assessed AlphaFold's model confidence score, which is a linear combination of pTM and ipTM (Evans et al., 2021) scores, as well as interface pLDDT (I‐pLDDT), which is based on residue‐level confidence scores for antibody–antigen interface residues (4 Å distance cutoff), as used in previous studies (Bryant et al., 2022; Yin et al., 2022), for discrimination of correct antibody–antigen models (Figure 3). While both exhibited significant correlations with DockQ score (Johansson‐Akhe & Wallner, 2022), which is a continuous measure of complex model accuracy, I‐pLDDT was marginally superior (Figure 3a,b); this was also evident for comparison of the scores with CAPRI accuracy levels (Figure 3c,d).

I‐pLDDT also provided outstanding discrimination between incorrect versus medium or higher accuracy models based on receiver operating characteristic (ROC) area under the curve (AUC) metrics (AUC = 0.92), which is higher than that of the model confidence (AUC = 0.88; Table 1). We also tested the individual components of the model confidence scores (pTM and ipTM) (Figure S9), which did not yield improved correlations with DockQ scores versus model confidence. When excluding data points without side‐chain contacts within 4 Å across the antibody–antigen interface (for which I‐pLDDT was set to an arbitrary minimum value in Figure 3b and in the corresponding correlation calculation), the correlation between the interface pLDDT and DockQ increased to *r* = 0.57 (Figure S10a), which demonstrates a more significant difference compared to the correlation between the model confidence and DockQ (*r* = 0.53; Figure S10b).

**TABLE 1:** Area under the ROC curve (AUC) value for protein model quality classes as a function of different scoring metrics.

Score^a^	Binary classification ROC AUC^b^		Multi‐class classification^b^
	Incorrect versus high	Incorrect versus medium and high	
Interface pLDDT	1.00	0.92	0.88
Model confidence	0.99	0.88	0.85
ipTM	0.99	0.87	0.85
pTM	0.99	0.88	0.84

^a^ Scoring methods. Model confidence, ipTM, and pTM are confidence scores from AlphaFold. Interface pLDDT is the average AlphaFold pLDDT score of antibody–antigen interface residues within 4 Å distance cutoff. Models without antibody–antigen interface contacts were assigned an interface pLDDT value of 30.

^b^ The ROC AUC values of binary classification and multi‐class classification were calculated using the R pROC (Robin et al., 2011) and multiROC (Wei & Wang, 2018) packages, with classes defined by model CAPRI accuracy, which assigned antibody–antigen models into incorrect (*n* = 9062), acceptable (*n* = 773), medium (*n* = 684), and high (*n* = 156) accuracy categories.

One advantage of I‐pLDDT over ipTM and model confidence (which primarily consists of ipTM) is that it is specifically focused on the antibody–antigen interface, whereas ipTM is calculated across all inter‐chain interfaces of complex models, including heavy–light and multiple antigen chains, thus the latter scores may be influenced by less relevant elements of the complex. Overall, these results support the use of I‐pLDDT as a primary metric in assessing the quality of AlphaFold antibody–antigen models.

### 2.4 Progressive improvements over recycling iterations

**FIGURE 4:**
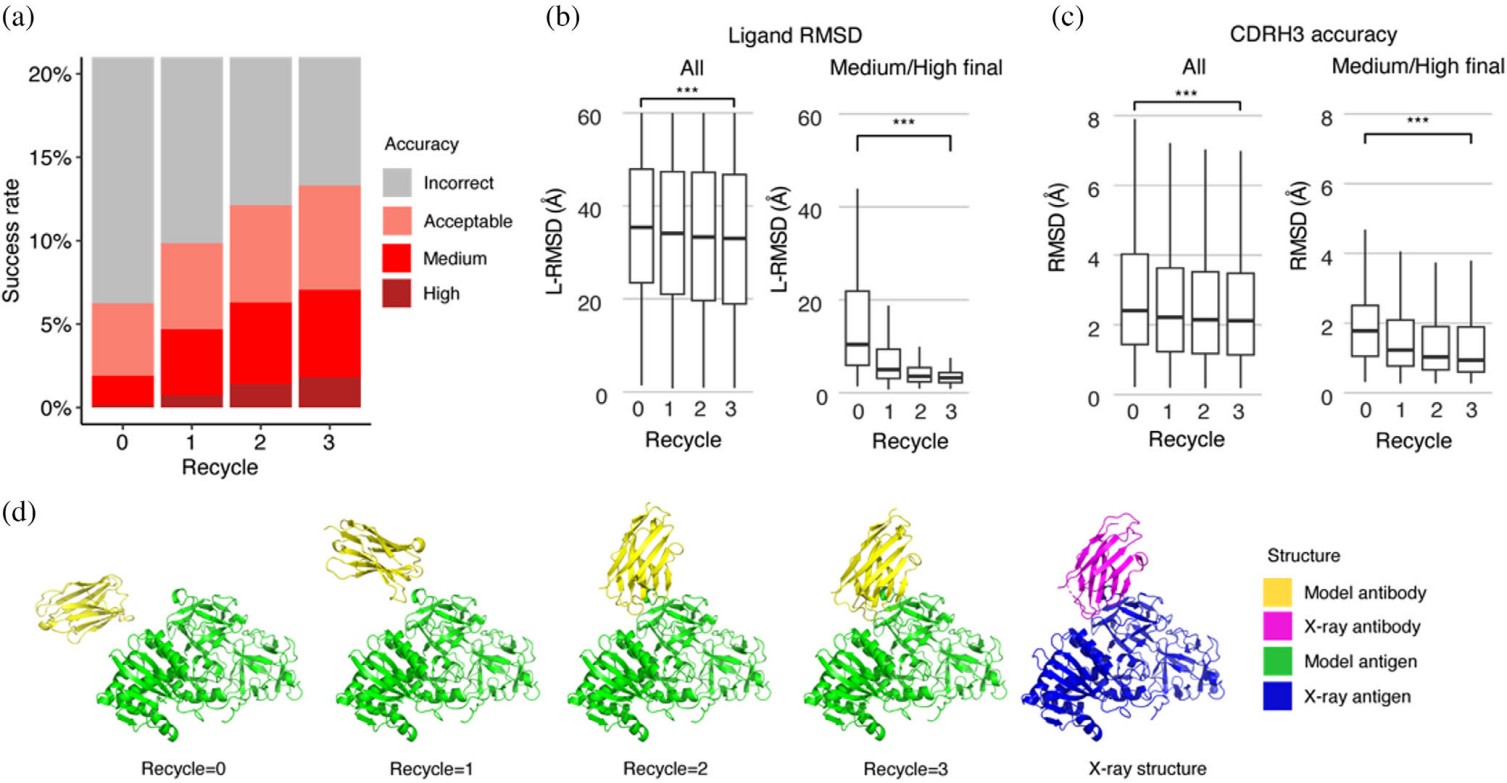
Analysis of antibody–antigen predictions across recycling iterations. (a) The accuracy of antibody–antigen complex predictions across up to three recycling iterations. Complex prediction accuracy across recycling iterations (up to three recycles, denoted by the *x*‐axis). Success rate is defined as the proportion of predictions of specific level of CAPRI criteria in a total of 25 prediction per complex, 426 complexes total, at the given recycle. Recycle = 0 denotes the state of the prediction before recycling iterations begin. (b) Distribution of the ligand RMSD (L‐RMSD, Å) of antibody–antigen prediction at each recycling iteration (denoted by the *x*‐axis), of all predictions (25 predictions × 426 complexes, left panel) or a subset of predictions of medium or high CAPRI accuracy at recycle = 3 (106 predictions, right panel). (c) Distribution of the CDRH3 accuracy of antibody–antigen prediction at each recycling iteration (denoted by the *x*‐axis), of all or a subset of predictions of medium or high CAPRI accuracy at recycle = 3. CDRH3 accuracy is defined as the change in RMSD of the CDRH3 region, when superposing the predicted antibody (in the antibody–antigen complex prediction) onto the experimentally resolved antibody (in the antibody–antigen complex) using the antibody framework region. Statistical significance values (Wilcoxon rank‐sum test) were calculated between RMSD values for sets of predictions at the outset of recycling iterations (recycle = 0) versus at recycle = 3, as noted at top (***p ≤ 0.001). (d) Example of a prediction across recycling iterations (PDB 7kd2; nanobody/Ricin complex). This prediction's CAPRI accuracy level across recycles was incorrect at recycle = 0 (I‐RMSD = 17.98 Å), incorrect at recycle = 1 (I‐RMSD = 10.90 Å), acceptable at recycle = 2 (I‐RMSD = 2.52 Å), and medium at recycle = 3 (I‐RMSD = 1.45 Å). The CDRH3 RMSDs of the predictions across recycling iterations 0, 1, 2, and 3 were 1.39 Å, 1.19 Å, 1.27 Å, and 1.17 Å, respectively. The L‐RMSDs of the predictions across recycling iterations 0, 1, 2, 3 were 49.95 Å, 24.68 Å, 5.42 Å, 4.25 Å, respectively. Antibody and antigen chains of the predictions and *x*‐ray structure are colored as indicated. Predictions were generated with ColabFold due to its faster model generation speed compared to AlphaFold.

Recycling is a critical component of the AlphaFold algorithm (Evans et al., 2021; Jumper et al., 2021a), wherein each model is input back to the system for further optimization. To improve our understanding of the impact of recycling iterations on AlphaFold modeling of antibody–antigen complexes, we modified the AlphaFold pipeline in ColabFold. ColabFold was preferable to utilize in this context versus the default AlphaFold pipeline due to its speed, in order to enable output and analysis of the antibody–antigen complex predictions at each recycling iteration. Our analysis demonstrates an increase in model accuracy as recycling iterations progress (Figure 4a). In fact, approximately 50% of predictions of medium or higher accuracy after the third recycling iteration were incorrect models before recycling iterations (Figure S11).

Next, we analyzed specific changes in antibody–antigen model across recycling iterations, identifying notably enhanced features and those that are unchanged. Features that were improved highlights the strength of AlphaFold, whereas the lack of improvement may highlight areas of difficulty or suggest that these features were already optimal at the start and did not require further refinement. We analyzed both the accuracy of antibody positioning on the antigen and the quality of the highly variable CDR loop of the antibody. Given the high variability in CDRH3 RMSD (Figure S6), compared to the RMSD of other CDR loops, we focused our analysis of CDR loops on the CDRH3. Considering all predictions, we observed a marginal yet significant improvement in both the antibody–antigen binding conformation as measured by ligand RMSD (L‐RMSD) (Figure 4b, left panel) and the CDRH3 loop accuracy (Figure 4c, left panel). Upon examining the subset of cases with medium or higher accuracy at recycle 3, we observed that the antibody–antigen binding conformation score L‐RMSD exhibited a pronounced and significant improvement (Figure 4b, right panel), while the improvement in CDRH3 loop RMSD was significant but not as pronounced (Figure 4c, right panel), indicating that for models to attain high accuracy at the end of the recycling iteration, it is helpful for AlphaFold to accurately predict the CDRH3 loop relatively accurately before recycling iterations begin.

The capability of AlphaFold to perform rigid‐body protein movements over recycling iterations, is shown in Figure 4d (nanobody/Ricin complex). This prediction was of incorrect accuracy before recycling iterations and was improved to a medium accuracy prediction at Recycle 3. Over recycling iterations, the L‐RMSD of this prediction exhibited a substantial degree of improvement, from 49.95 Å before recycling, to 4.25 Å at recycle 3. Unlike L‐RMSD, the CDRH3 loop of this prediction was accurately predicted (CDRH3 RMSD = 1.39 Å) before the recycling iterations.

The importance of CDRH3 loop accuracy for complex modeling success was further explored by the analysis of CDRH3 loop conformations of modeled unbound structures. Unbound antibody structures were generated with AlphaFold with a template date cutoff of April 30, 2018, and the CDR loops of the unbound antibody models were compared to those of the antibodies in the antibody–antigen complexes. The RMSD between CDR loops of the unbound models and the antibody in the bound is compared against the complex modeling success of top‐ranked antibody–antigen models generated by AlphaFold in Figure S12. Although the relatively small numbers of high accuracy cases limit this comparison, the accuracy of the CDRH3 modeling in unbound antibody structures for high antibody–antigen models was found to be significantly higher than that of the incorrect accuracy models (*p* ≤ 0.05), suggesting that antibodies with unbound models that more closely resemble the bound loop conformation are likely to be more accurately modeled in the form of antibody–antigen complexes.

### 2.5 Input of subunit chains in bound conformation enables higher success

**FIGURE 5:**
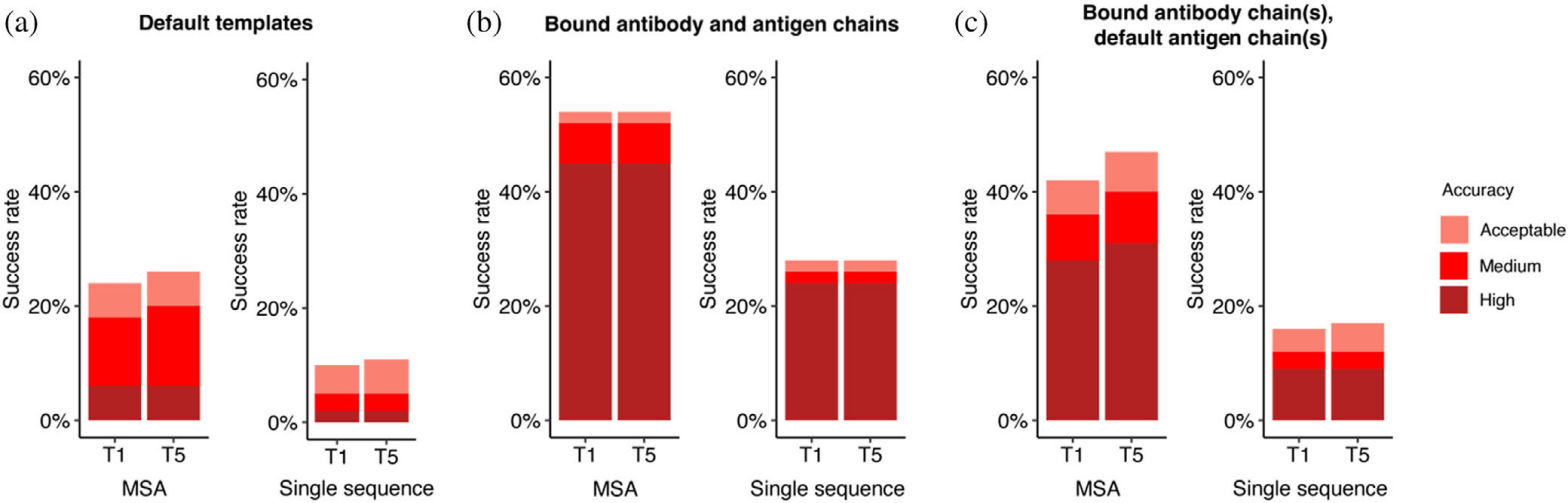
Improved subunit modeling enhances antibody–antigen complex modeling success. Antibody–antigen modeling success of AlphaFold by utilizing (a) templates identified through the default template search protocol, (b) bound antibody and antigen chains as templates, (c) bound antibody and default antigen chains (identified by the default search protocol) as templates. Benchmarking was performed on a total of 100 antibody–antigen complexes. The success rate was calculated based on the percentage of cases that had at least one model among their top *N* predictions that met a specified level of CAPRI accuracy. Bars are colored by CAPRI accuracy criteria.

To better understand the factors that can enhance the success rate of the AlphaFold antibody–antigen modeling, we utilized native antibody–antigen chains as templates within the AlphaFold modeling pipeline, to gauge whether AlphaFold can better assemble the complex structures given the bound subunit chains. Modifications were made to the AlphaFold pipeline to optionally input specific selected PDB templates for each chain. To test performance, we randomly selected 100 cases from the full antibody–antigen benchmark that do not have observed glycans at the antibody–antigen interface and do not belong to the partial antigen assembly category, due to observed change in performance for those sets of cases (Figure 2; Figure S6). On this subset of 100 cases, the use of default templates identified from the AlphaFold pipeline resulted in 18% success in generating near‐native (medium or high accuracy) top‐ranked predictions (Figure 5a), which is similar to the performance on the full benchmark (Figure 1a).

A substantial improvement in accuracy was observed when experimentally determined antibody–antigen chains were used as individual chain templates, in which case the success in generating near‐native top‐ranked predictions was 52% (Figure 5b). Analysis of the top‐ranked prediction success determinants shows that distribution of interface energy score (Figure S13a) and change in solvent‐accessible surface area (ΔSASA) for hydrophobic part of the antibody–antigen interface (Figure S13b) are significantly different (*p* ≤ 0.01) between complexes that have incorrect versus high accuracy top‐ranked predictions, indicating that despite using bound template structures, AlphaFold has difficulty predicting the complex structure for antibody–antigen interactions with less favorable computed interface energies and with smaller hydrophobic interface area. Using only subsets of experimentally determined antibody–antigen chains as templates, as well as use of experimentally resolved antigens bound to other antibody structures, resulted in a decrease in model accuracy, compared to using all experimentally determined chains as templates (Figures S14 and S15; Supplementary Results). Interestingly, rigid‐body docking in ZDOCK with bound component inputs achieved comparable, although moderately higher, medium/high accuracy success compared to AlphaFold with bound component templates (Figure S14a), indicating that both rigid‐body docking and deep learning can both perform antibody–antigen complex assembly from bound components, albeit not in all cases (~50%–60% medium/high accuracy success for top‐ranked models).

### 2.6 MSA provides important information for accurate prediction of complexes

We also evaluated the performance of AlphaFold without MSAs, to test the impact on complex assembly when subunit structures are known (thus MSA would not in principle be needed for subunit structure modeling), given the likely lack of direct co‐evolutionary information present in antibody–antigen MSAs. The removal of MSAs was implemented through modifications to the AlphaFold pipeline, as noted in the Section 4. Our results indicated a notable decrease in accuracy when MSA was disabled, as compared to the with‐MSA counterparts (Figure 5; Figure S14b,c). This prompted us to investigate the possible association between the depth of MSA and the modeling outcome by AlphaFold.

**FIGURE 6:**
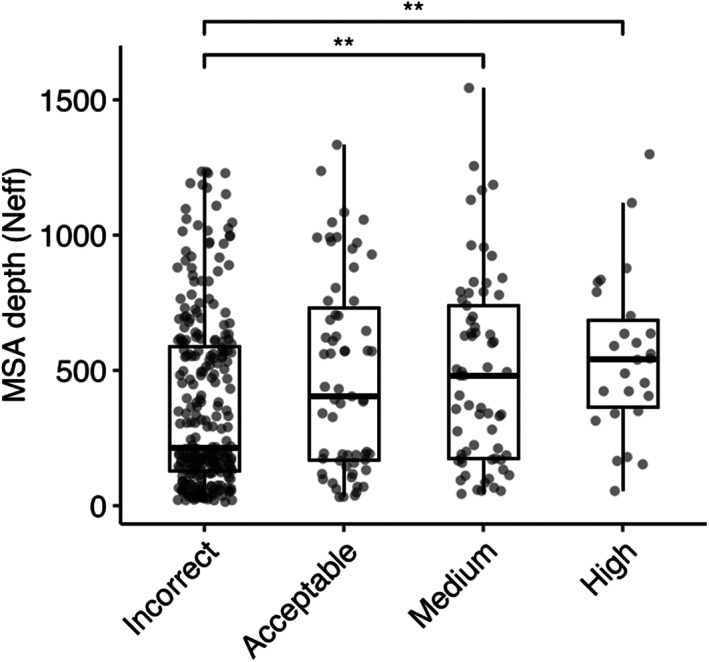
Comparison of MSA depth and modeling success. The distribution of MSA depth (number of effective sequences, N_eff_), calculated using CD‐Hit (Fu et al., 2012) with an identity cutoff of 80%, is shown for antibody–antigen complexes grouped by AlphaFold modeling accuracy. The modeling accuracy is defined as the highest CAPRI criteria prediction in the complex, considering all 25 predictions. Numbers of data points in incorrect, acceptable, medium and high categories are 272, 63, 65 and 26. Statistical significance values (Wilcoxon rank‐sum test) were calculated between interface energy scores for sets of cases with incorrect versus medium and incorrect versus high CAPRI accuracy predictions, as noted at top (**p ≤ 0.01).

We investigated the impact of MSA depth on modeling success by the full AlphaFold protocol, grouping the complexes by prediction accuracy and comparing distributions of MSA depth (N_eff_) (Figure 6). The distribution of N_eff_ was found to be statistically significant between incorrect and medium accuracy classes (*p* ≤ 0.01), and between incorrect and high accuracy classes (*p* ≤ 0.01). We also compared the docking model quality (DockQ score) for all cases when binned by MSA depth levels (Figure S16). A slight trend was observed indicating that a greater MSA depth is associated with higher DockQ scores (higher model accuracies), suggesting that compared to a shallow MSA, predictions with a deeper MSA are more likely to be of higher accuracy. Thus, it is possible that increasing MSA depth, particularly for antibody–antigen complexes with very shallow MSAs, could lead to some improvement in overall modeling performance.

### 2.7 Modeling accuracy of AlphaFold v.2.3.0

**FIGURE 7:**
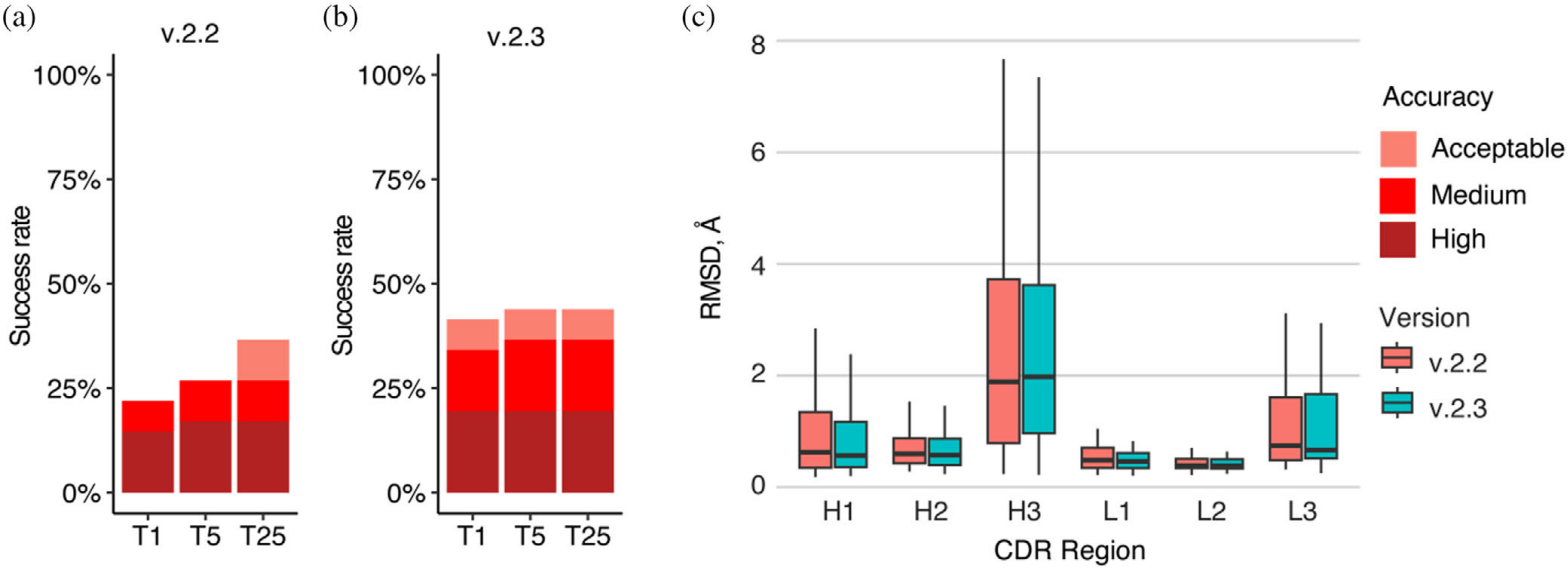
Antibody–antigen modeling success by AlphaFold v.2.3. Modeling success of (a) AlphaFold v.2.2 and (b) AlphaFold v.2.3 on 41 antibody–antigen complexes. The success rate was calculated based on the percentage of cases that had at least one model among their top *N* predictions that met a specified level of CAPRI accuracy. Bars are colored by CAPRI accuracy criteria. (c) Distribution of the CDR loop prediction accuracy of AlphaFold v.2.2 (denoted by salmon color) versus v.2.3 (denoted by cyan color). CDR loop accuracy is defined as the change in RMSD of the CDR regions, when superposing the predicted antibody (in the antibody–antigen complex prediction) onto the experimentally resolved antibody (in the antibody–antigen complex) using the antibody framework region.

Recently, an updated version of AlphaFold (v.2.3.0, hereafter denoted as v.2.3) was released, with modifications to the pipeline and deep learning model (DeepMind, 2022). Compared with the previous version, this version was trained on PDB structures released until September 30, 2021, resulting in a 30% increase in training data. This version also increased the maximum number of recycles, from 3 recycles in v.2.2 to 20 recycles in v.2.3, with early stopping, and utilized larger interface regions (crops) and more chains during training. To benchmark its performance, we assembled a test set of 41 nonredundant antibody–antigen complexes released after the September 30, 2021 training date (Table S1). AlphaFold v.2.3 generated medium or higher accuracy models as top‐ranked predictions for 36% of the test cases, notably higher than the 23% generated by v.2.2 (Figure 7), with no significant difference in antibody CDR loop accuracy. Additional benchmarking revealed that reducing the number of recycling iterations in v2.3 to match the number of recycling iterations in v2.2 resulted in unchanged modeling success for v2.3 (Figure S17; Supplementary Results). This suggests that the observed difference in the number of recycles between v.2.3 and v.2.2 is not the main factor contributing to the increased success, and that the updated and expanded training of the deep learning model training may be responsible.

**FIGURE 8:**
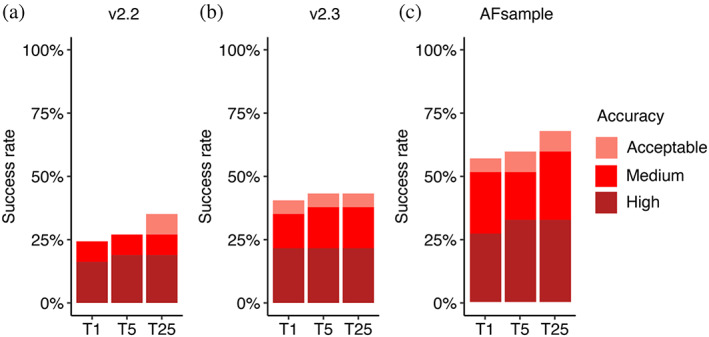
Antibody–antigen modeling success by AlphaFold v.2.2, v2.3 and AFsample. Modeling success of (a) AlphaFold v.2.2, (b) AlphaFold v.2.3, and (c) AFsample on 37 antibody–antigen complexes. The success rate was calculated based on the percentage of cases that had at least one model among their top *N* predictions that met a specified level of CAPRI accuracy. Bars are colored by CAPRI accuracy criteria.

A recent study demonstrated that by introducing stochastic perturbations through activating dropout during AlphaFold inference and employing extensive sampling, the modeling success of AlphaFold can be improved (Wallner, 2023a). Using this technique, named AFsample, the Wallner group ranked among the top predictors in CASP15 for protein assembly modeling, which included five nanobody‐antigen and three heavy–light antibody–antigen targets (Lensink, Brysbaert, Raouraoua, Bates, et al., 2023; Wallner, 2023b). In light of this, we applied the AFsample protocol to model our benchmarking set of antibody–antigen complexes to assess its performance on a broader dataset. On a total of 37 cases for which all models were successfully generated, AFsample generated medium or higher accuracy top‐ranked predictions for 51% of the test cases, which is notably higher than 35% for AlphaFold v.2.3, and 24% AlphaFold v.2.2 (Figure 8). When the top 25 predictions were considered, AFsample's medium or higher success rate increased to 59%. In summary, our findings indicate that massive sampling through a combination of dropout, pooling structures from different AlphaFold models and parameters, and generation of large numbers of models, provides a clear advantage over the standard protocol in the context of antibody–antigen complex modeling, although with substantially higher computational cost.

## 3 DISCUSSION

Using a set of over 400 nonredundant antibody–antigen complexes, we benchmarked and evaluated AlphaFold's ability to model antibody–antigen complexes. On this set, we observed a limited yet higher success in the prediction of antibody–antigen structures by AlphaFold, compared to our previous benchmarking that used an older AlphaFold version accessed via ColabFold, and was based on a limited set of 100 antibody–antigen cases (Yin et al., 2022). Analyses of factors that could influence the prediction outcome showed that AlphaFold was less able to accurately predict antibody–antigen structures with glycans at the antibody–antigen interface, which highlights AlphaFold's limitation in handling complexes with post‐translational modifications. We also found that AlphaFold is more successful at modeling nanobody–antigen complexes and has difficulty predicting the structure of larger antibody–antigen complexes. An analysis of prediction accuracy at each recycling iteration, as well as the bound antibody–antigen template tests shows the importance of accurate subunit modeling for success in predicting the antibody–antigen complex. Relatedly, the ability to accurately predict CDRH3 loops is important for overall docking success.

Our benchmarking also shows that the latest version of AlphaFold (v.2.3) exhibits improved success in predicting antibody–antigen structures versus the previous AlphaFold version (v.2.2), likely due at least in part to the model training on an updated and expanded set of complex structures from the PDB (DeepMind, 2022). It is possible that success can be improved further through additional optimization or other adaptations of the AlphaFold framework or model. Additionally, we observed that a recently described AlphaFold‐based massive sampling approach, named AFsample (Wallner, 2023a), achieved even higher success than standard AlphaFold 2.3. It is possible that additional sampling, or pooling with different sets of models and parameters, could improve this success further. Another potential avenue for elevating the accuracy of AlphaFold predictions is demonstrated by the recent development of fully trainable AlphaFold implementations (Gustaf et al., 2020; Motmaen et al., 2023; Ziyao et al., 2008), which enable researchers to adapt and refine the model to specific datasets or domains of interest, opening up new possibilities for customization and optimization of the AlphaFold network.

Despite the lack of explicit coevolutionary signal, our data show that the inclusion of diverse sequence information in MSAs is helpful for maintaining AlphaFold's modeling success of antibody–antigen complexes. As such, curation or optimization of MSAs could be another avenue for improving the accuracy of AlphaFold predictions. Previous work showed that AlphaFold prediction of protein–protein complexes can be augmented with improved MSA cross‐chain pairing (Bryant et al., 2022), while others have developed alternative MSA methods such as DeepMSA2 (Zheng et al., 2021), which was part of a successful pipeline in a recent CASP/CAPRI complex structure prediction round (Lensink, Brysbaert, Raouraoua, et al., 2023). Recent work leveraging protein language models shows promise in constructing diversified and informative MSAs for enhancing accuracy in AlphaFold protein complex prediction (Bo et al., 2009), while it may be possible to replace or augment the MSA in AlphaFold with language model representations, potentially building on recent language models developed for antibodies (Olsen et al., 2022; Ruffolo et al., 2021) or proteins in general (Hie et al., 2023).

Our results also demonstrate that accurate subunit prediction is associated with higher antibody–antigen complex prediction success. Recent work has shown improved accuracy in antibody prediction, particularly in the context of CDR loops, leveraging elements of AlphaFold architecture, especially the structure module, with modifications (Abanades et al., 2023; Ruffolo et al., 2023). Incorporating such advances into the prediction pipeline may enable the prediction of more accurate antibody–antigen complexes.

While it is possible or even likely that antibody–antigen modeling success may ultimately be improved in AlphaFold or related deep learning frameworks, the current success of AlphaFold, particularly when using its updated model (v.2.3) or a recently described massive sampling protocol, in conjunction with the observed confidence scoring accuracy, indicates that AlphaFold may potentially be of practical use to researchers in modeling this important and challenging class of complexes, and can complement or assist experimental structural determination methods.

## 4 METHODS

### 4.1 Antibody–antigen benchmark assembly

We assembled two nonredundant sets of high resolution structures to benchmark AlphaFold, following the general protocol that we described previously (Yin et al., 2022). To obtain an initial list of antibody–antigen complexes from the PDB, we downloaded the full SAbDab (Dunbar et al., 2014) antibody structure dataset in January 2022. The antibody–antigen complex dataset for AlphaFold v2.2 benchmarking was assembled using the following criteria: (1) structure resolution ≤3.0 Å, (2) protein antigen in the structure (based on SAbDab annotation), and (3) nonredundant with antibody–antigen complexes with structural resolution ≤9.0 Å released before April 30, 2018 (AlphaFold v2.2 training sample cutoff date) based on sequence criteria. Sequence criteria for nonredundancy are: (1) heavy chain variable domain sequence ID <90% and full variable domain sequence ID <90%, or (2) no match between antigen chain sequences (no hit detected using BLAST (Camacho et al., 2009) with default parameters). Pairwise sequence alignments were performed using the “blastp” executable in the BLAST suite (Camacho et al., 2009). Structural nonredundancy criteria were then applied to the set. We removed antibody–antigen structures with <5 Å heavy chain Cα atom RMSD, after superposition of antigens using the FAST structure alignment program (Zhu & Weng, 2005), and > 70% identity between heavy chain variable domain, light chain variable domain, or concatenated CDR loop sequences. To avoid modeling antigen chains with large regions that are not resolved in the experimentally determined structures, we additionally removed structures with PDB “seqres” file sequence annotation and resolved region sequence length difference >70%, or sequence length difference >35% and resolved antigen length >500 aa. We also removed non‐canonical antibody–antigen complex cases (e.g., with antibody‐tetramerization, dimeric sdAb, or constant domain binding), and we removed cases with incomplete antigen chain annotations by SAbDab, identified through manual inspection of the PDB bioassembly structure.

To benchmark AlphaFold v.2.3, we identified a subset of 41 antibody–antigen complexes within the v.2.2 benchmarking set. These antibody–antigen complexes were released after September 30, 2021, and are not redundant with structures released before that date based on the sequence criteria detailed above. The AlphaFold v2.2 and v2.3 benchmarking cases are shown in Table S1.

### 4.2 AlphaFold antibody–antigen modeling

Sequences input to AlphaFold were obtained from the PDB “seqres” file. Antibody sequences were processed by ANARCI to remove non‐variable domain sequence regions. We downloaded and installed AlphaFold v2.2 from Github (https://github.com/deepmind/alphafold) in May 2022 and v.2.3 in February 2023. Both versions of AlphaFold were installed on a local computing cluster. During the structure prediction or feature preparation step in the AlphaFold pipeline, 15 cases failed to complete because of GPU and memory limitations out of a total of 442 test cases.

For generating unbound antibody and antigen structures, we employed AlphaFold in Multimer setting when the input consisted of a heavy‐light chain antibody or a multimeric antigen. Alternatively, the Monomer setting was utilized when the input was a single chain. A template date cutoff of April 30, 2018 was applied to avoid template overlap with benchmarking set.

To generate AlphaFold predictions without the use of MSAs (corresponding to single‐sequence modeling), we modified “all_seq_msa_features” variable of chain features, to include only the query sequence. To use custom templates, we adapted the template featurization function from Motmaen et al. (2023) (https://github.com/phbradley/alphafold_finetune/blob/main/predict_utils.py).

AlphaFold modeling in ColabFold (Mirdita et al., 2022) was performed with ColabFold version 1.4.0 (commit 26de12d3afb5f85d49d0c7db1b9371f034388395), installed on a local computing cluster using scripts from Github (https://github.com/YoshitakaMo/localcolabfold). During ColabFold AlphaFold modeling, MSA was built by querying the MMseqs2 MSA server using unpaired and paired MSA. To generate a total of 25 predictions per complex, modifications were made to “load_models_and_params” function, utilizing a different random seed for each prediction, producing five predictions per AlphaFold model parameter.

Unless otherwise specified, a template date cutoff of April 30, 2018 was applied for benchmarking AlphaFold v.2.2 and ColabFold, and a template date cutoff of September 30, 2021 was applied for benchmarking of AlphaFold v.2.3, to avoid using bound structures as template.

AlphaFold and ColabFold modeling runs were performed using NVIDIA Titan RTX and Quadro 6000 GPUs.

### 4.3 Complex model accuracy assessment

We assessed antibody–antigen complex model accuracy using DockQ (Johansson‐Akhe & Wallner, 2022), which was downloaded from GitHub (https://github.com/bjornwallner/DockQ). Antibody–antigen complex model accuracy was computed by DockQ using the experimentally determined antibody–antigen complex structures obtained from the PDB. DockQ calculates interface backbone RMSD (I‐RMSD), ligand backbone RMSD (L‐RMSD), fraction of native contacts (fnat), DockQ score, as well as the Critical Assessment of PRediction of Interactions (CAPRI) accuracy level, which assigns the model into one of four discrete accuracy classes: incorrect, acceptable, medium, and high, based on the model's similarity to the native structure (Lensink et al., 2020).

### 4.4 Interface pLDDT calculation

To determine the interface pLDDT (I‐pLDDT), we computed the average pLDDT value for all residues at the antibody–antigen interface. Interface residues were defined as any residue with a non‐hydrogen atom within 4.0 Å of the binding partner. An I‐pLDDT score of 30 was assigned to predictions with no antibody–antigen interface residues.

### 4.5 CDR loop accuracy analysis

The CDRs and the framework regions of antibodies were identified by AHo numbering (Honegger & Pluckthun, 2001), assigned using ANARCI software (Dunbar & Deane, 2016). The CDR loops were defined as residues 24–42 (CDR1), 57–76 (CDR2), and 107–138 (CDR3), as in previous work (Lee et al., 2022).

ProFit v 3.1 (Martin & Porter, 2009) was used to calculate backbone RMSDs between modeled and experimentally determined CDR loop structures, after superposing the modeled antibody structures onto the experimentally resolved structures by the framework residues.

### 4.6 Figures and statistical analysis

PyMOL (Schrodinger, Inc.) was used to generate structural figures. The ggplot2 (Wickham, 2016) package in R (r-project.org) was utilized to generate box plots, line plots, and bar plots. Pearson correlations and their corresponding *p* values were calculated using the ggpubr package in R, while the Wilcoxon rank‐sum test was performed using the ggsignif package in R. Binary and multi‐class ROC AUC values were calculated using the pROC (Robin et al., 2011) and multiROC (Wei & Wang, 2018) packages in R, respectively.

## AUTHOR CONTRIBUTIONS

**Brian G. Pierce:** Conceptualization; methodology; funding acquisition; writing – review and editing; supervision. **Rui Yin:** Conceptualization; methodology; writing – original draft; writing – review and editing; formal analysis; data curation.

## Supporting information


**DATA S1:** Supporting Information.

## Data Availability

Modified AlphaFold code and analysis scripts are available on Github: https://github.com/piercelab/alphafold_v2.2_customize. AlphaFold2.2, AlphaFold2.3, and ColabFold antibody–antigen models generated in this study are available for download at: https://piercelab.ibbr.umd.edu/af_abag_benchmarking.html.
