# Supplementary material for: Evaluation of AlphaFold antibody–antigen modeling with implications for improving predictive accuracy
Source: Protein Sci. 2024 Jan 1;33(1):e4865. doi: 10.1002/pro.4865 (PMC10751731; doi:10.1002/pro.4865)
Supplement: Supplementary file 1 — DATA S1: Supporting Information. [file PRO-33-e4865-s001.pdf]

Supporting Information for

Evaluation of AlphaFold antibody-antigen modeling with implications for improving predictive accuracy

Rui Yin and Brian G. Pierce

Table of Contents

Supplementary Methods **3**
Antibody-antigen complex scoring and native complex relaxation 3
Rigid-body docking with ZDOCK and IRAD 3
Rigid-body docking with ClusPro 4
TM-score calculation 4
MSA depth calculation 5
Hetero-atoms at the interface 5
AFsample antibody-antigen modeling 7
Identification of experimentally resolved antigens bound to other antibodies 7
Supplementary Results **9**
Comparing AlphaFold with rigid-body docking algorithms 9
Additional antibody-antigen modeling success determinants 10
Accurate subunit modeling and antibody-antigen prediction success 12
Utilizing antigen structures bound to other antibodies as templates 13
Sustained v2.3 modeling success despite fewer recycles 14
Supplementary Figures **15**
Supplementary Table **32**
References **44**

Supplementary Methods

Antibody-antigen complex scoring and native complex relaxation

The "InterfaceAnalyzer" executable in Rosetta[1] (v.3.12) was employed to calculate interface energetic scores, using the Rosetta Energy Function 2015 (REF15) scoring function[2] and default parameters. Prior to scoring, structural relaxation was performed on native antibody-antigen complex obtained from PDB using the FastRelax protocol[3] ("relax" executable) in Rosetta using the following flags:

-ignore_unrecognized_res

-relax:constrain_relax_to_start_coords

-relax:coord_constrain_sidechains

-relax:ramp_constraints false

-ex1

-ex2

-use_input_sc

-no_optH false

-flip_HNQ

-overwrite

-nstruct 1

Rigid-body docking with ZDOCK and IRAD

To establish a rigid-body docking baseline, ZDOCK version 3.0.2[4] was used to generate antibody-antigen docking models using unbound or bound input structures. Unbound antibody and antigen input structures for the ZDOCK algorithm were generated by AlphaFold, using the

above-mentioned protocol. Bound antibody and antigen input structures were extracted from experimentally resolved antibody-antigen complex structures downloaded from PDB, with HETATMs removed. Through dense rotational sampling ("-D" flag in ZDOCK), a total of 54,000 predictions per complex were generated. Subsequently, the docking poses were scored and reranked using the IRAD scoring function[5]. Predictions were assessed with the DockQ program[6] to determine the CAPRI model accuracy based on comparison with the known complex structure.

Rigid-body docking with ClusPro

To establish a baseline for a rigid-body docking algorithm specific to antibody-antigen complexes, the ClusPro web server (https://cluspro.bu.edu/) was utilized in its antibody mode[7]. Unbound antibody and antigen input structures (same input for unbound ZDOCK docking) were generated by AlphaFold as described above. Within the ClusPro interface, the options 'Use Antibody Mode' and 'Automatically Mask non-CDR regions' were selected, in accordance with the server's recommendations. Docking poses were ranked using ClusPro's default method, based on cluster size from large to small. All docking poses were assessed with the DockQ program[6] to assess CAPRI model accuracy based on comparison with the known complex structure.

TM-score calculation

To provide an assessment of antigen prediction accuracy, we employed the TM-score executable[8] to calculate the TM-score, comparing the structural similarity between the antigen chain(s) in the experimentally resolved antibody-antigen complex structure, and the antigen prediction in the antibody-antigen complex predictions. Prior to running TM-score on the antigen chains, residues that were not experimentally resolved, or absent from the experimentally resolved structure, were removed from the antigen prediction.

MSA depth calculation

The MSA of the antibody-antigen complex was retrieved from the 'msa' key value in the feature dictionary ("feature_dict" variable). Given that the MSA values of the 'msa' key are encoded in AlphaFold residue IDs, we converted the amino acids back to the one-letter amino acid type using "ID_TO_HHBLITS_AA" dictionary, and replaced gaps (denoted by "-") by "U" for downstream MSA depth calculation. Number of effective sequences (N_{eff}) was calculated by CD-Hit[9]. For consistency with the AlphaFold MSA N_{eff} calculation scheme[10], we used an identity cutoff of 80% in CD-HIT to calculate nonredundant sequence clusters. MSA depth was successfully calculated for 426 out of 427 antibody-antigen complexes, with one failure due to a technical issue.

Hetero-atoms at the interface

Glycans and ligands at the antibody-antigen interface were identified through inspection of HETATM records in experimentally resolved structure coordinates. Asymmetric unit structures (defined by the PDB entry) of the antibody-antigen complexes were inspected, and cases with hetero-atoms matching the "saccharide" classification identified from PDB ligands summary pages (from wwPDB's Chemical Component Dictionary), as well as hetatoms in the category of lipids and nucleotides within 6 Å of the antibody and the antigen were identified as positive hits.

Glycan clusters were also accounted for, in order to appropriately group individual glycan residues into a single N-glycan during analysis. Glycan clusters are identified by checking the distance between the C and the O atoms of glycan HETATM residues "MAN", "BMA", "NAG", and "FUC". Glycans were considered to be covalently linked if their C atom to another glycan residue's O atom has a distance < 2.0 Å. One cluster of glycans is formed by pooling together all glycan residues that are covalently attached. One cluster of glycans were considered

in the interface between antibody and antigen, if the cluster has glycan residues < 6 Å to non-hydrogen atoms of antibody chains, and has glycan residues < 6 Å to non-hydrogen atoms of antigen chains.

Since not all antigen glycosylation is experimentally resolved, we predict the possible presence of antigen glycosylation near antibody binding site based on the source species of the antigen and the presence of surface-accessible glycosylation motifs. We first determined the source species of each antigen in our dataset to determine if the antigen can be glycosylated. This assessment was based on whether proteins from these species are known to undergo glycosylation either intrinsically or through the hijacking of host machinery, as observed in enveloped viruses that infect eukaryotic hosts. In our set, such species include eukaryotic species (excluding Plasmodia, Butyriboletus subregius, Atractiella rhizophila, Aequorea victoria, Betula pendula due to uncertain, less predictable, or lack of N-glycosylation for those organisms) and enveloped viruses. For antigens identified as possible to be N-glycosylated based on source organism, we next examined their sequences for antibody-proximal glycosylation motifs. Specifically, we focused on N-glycosylation sequons, defined as the sequence motif N-X-S/T (where X is any amino acid except proline). We then employed Naccess v2.1.1[11] to evaluate the surface accessibility of the asparagine residue in each sequon. We consider those with a relative accessibility of the side chain above 15% as surface-exposed asparagine residues. Finally, we measured the proximity of these candidate asparagine residues to the antibody in the antibody-antigen complex structure, classifying those within 12 Å as potential sites of antigen glycosylation near the antibody binding site.

AFsample antibody-antigen modeling

AFsample was downloaded from GitHub (https://github.com/bjornwallner/alphafoldv2.2.0). The modeling protocol described in Wallner, 2023[12] was followed. A total of 6,000 predictions were generated per case, including 2,000 predictions generated using AlphaFold v.2.1 and v.2.2 models with templates and full dropout, 2,000 predictions generated using v.2.1 and v.2.2 models without templates and with dropout in Evoformer but not structure module, 1,000 predictions generated with v.2.1 models without templates, with maximum number of 21 recycles, with dropout in the Evoformer but not the structure module, and 1,000 predictions generated with v.2.2 models without templates, with maximum number of 9 recycles, with dropout in the Evoformer but not the structure module. When templates were used, a template date cutoff of September 30, 2021 was applied. The top 5 predictions per case were relaxed. Modeling runs were performed using NVIDIA Titan RTX, Quadro 6000 and RTX A100 GPUs.

Identification of experimentally resolved antigens bound to other antibodies

Antigen chains bound to distinct antibodies were identified through a systematic approach. Each antigen sequence from the complexes was queried against the PDB SEQRES database using BLAST[13] to find PDB chains with at least 95% sequence identity, covering a minimum of 90% of the query antigen sequence and structural resolution <= 3.0 Å. Subsequently, the SAbDab[14] database was utilized to determine if the identified PDB chains were complexed with antibodies. The hits were further examined for antibody distinctness from the test case. Antibodies complexed with the antigen hit were considered distinct if the heavy chain V domain sequence identity with the query antibody heavy chain was < 90%, or the full antibody V domain sequence identity was < 90%. We additionally removed antigen hits where the experimentally resolved sequence covered < 70% of the query antigen sequence. Finally, all qualifying hits were ranked

by their resolution, from lowest to highest, and the top hit was selected as the template for modeling.

Supplementary Results

Comparing AlphaFold with rigid-body docking algorithms

To compare antibody-antigen modeling performance with a previously developed docking approach, we utilized the global rigid-body docking algorithm, ZDOCK (version 3.0.2)[4], with the IRAD (Integration of Residue- and Atom-based Potentials for Docking) reranking function which was developed to improve the ranking of near-native ZDOCK models[5]. Since many of the antibody-antigen complexes in our benchmark set do not have experimentally determined unbound antibody and/or antigen structures, AlphaFold was employed to generate unbound antibody and unbound antigen inputs for ZDOCK, using templates released on or before April 30, 2018. We selected the top-ranked prediction from AlphaFold as the input for ZDOCK, and only performed ZDOCK docking with unbound structures having a minimum average pLDDT score over 80, in order to exclude cases with likely low quality input modeled structures. In total, 389 complexes met the criteria for minimum average pLDDT score cutoff. Using ZDOCK with IRAD reranking, the majority of test cases did not yield highly accurate predictions (1% medium or higher accuracy, **Fig. S2a**) as the top-ranked prediction. In contrast, AlphaFold-generated antibody-antigen complex models have a higher percentage of cases (19%) with medium or higher accuracy top-ranked predictions within this set (**Fig. S2b**). The success rate increases when we consider top 25 predictions generated by ZDOCK (7% of complexes have medium or higher accuracy predictions, **Fig. S2a**), yet the success is still lower than AlphaFold, which produced medium or higher accuracy predictions for 23% of cases when all 25 predictions were considered. This ZDOCK success is similar to the unbound antibody-antigen docking success in Guest et al.[15], although the difference in test cases, inputs (unbound versus

modeled structures), and ZDOCK sampling and model scoring do not support a direct comparison of success rates.

To compare AlphaFold with a rigid-body docking algorithm that is specialized in antibody-antigen docking, we additionally generated antibody-antigen docking predictions using ClusPro in antibody mode[7] on a subset of 100 cases randomly subsampled from the 389 ZDOCK test set cases. As with ZDOCK, the input for ClusPro consisted of the unbound antibody and antigen models produced by AlphaFold, which met the quality criteria as previously described. The default ranking method of the ClusPro server, which is based on the cluster size, was employed for evaluating the docking poses. ClusPro generated comparable results as ZDOCK. On this set, AlphaFold generated medium or higher accuracy top-ranked predictions for 19% of the test cases (**Fig. S3a**), whereas ZDOCK achieved this in 1% of cases (**Fig. S3b**), and ClusPro did not produce any such top-ranked predictions (**Fig. S3c**). When top 20 predictions were considered, the success rates rose to 24% for AlphaFold (**Fig. S3a**), 6% for ZDOCK (**Fig. S3b**) and 9% for ClusPro (**Fig. S3c**).

Challenges for the rigid-body docking algorithms likely responsible for the relatively low observed success rates include possible local inaccuracies in the unbound models, binding conformational changes (e.g. in CDR loops) even in the case of ideal unbound models, as well as modeled flexible protein terminal regions that are not resolved in some of the structures, which can lead to false positive binding sites for ZDOCK and ClusPro models.

Additional antibody-antigen modeling success determinants

While all complexes were modeled with antigen chains comprising the full epitope, a subset of cases did not include additional chains from the full antigen multimeric assembly (based on PDB "bioassembly") in the modeling, due to computational limitations and for

modeling efficiency. We examined whether these "partial antigen assembly" cases (N=49) were not as successfully modeled as the non-multimer or full assembly cases (N=378, **Fig. S6a**), due for instance to non-native surface regions that are normally buried in the full antigen assembly being engaged by antibodies in the models. Indeed, the partial antigen assembly cases exhibited lower modeling success versus the remainder of the cases. Additionally, consistent with our previous benchmarking study[16], success analysis on the current set of antibody-antigen complexes shows that larger complexes are generally more difficult to predict (**Fig. S6b**).

We also examined the accuracy of six complementarity determining region (CDR) loops in the modeled complexes, computing CDR loop RMSDs in the models with respect to the corresponding CDR loop from the experimentally determined complex structure (**Fig. S7**). Interestingly, the CDRH3 loop exhibited differences in accuracy across sets of models with different CAPRI accuracy levels, with median CDRH3 RMSD of 0.6 Å for high accuracy models, 1.2 Å for medium accuracy models, 2.2 Å for acceptable accuracy models, and 2.4 Å for incorrect models, indicating that accurate prediction of CDRH3 loops is associated with near-native modeling accuracy in antibody-antigen complex prediction in AlphaFold. However, it should be noted that CDR loop accuracy (among other features of the modeled interfaces) can play a role in the CAPRI antibody-antigen complex accuracy assessments themselves, with high CAPRI accuracy models likely requiring relatively low CDRH3 RMSDs to closely reflect the native interface.

We also examined possible failures of antigen structure modeling as factors in antibody-antigen modeling success (**Fig. S8**). The analysis of the antigen accuracy in top-ranked complex predictions revealed that while only 12 complexes out of 427 complexes have antigen predictions with TM-score[8] values below 0.7 with respect to the experimentally determined

antigen, indicating a relatively lower level of structural similarity[17], the majority of complex predictions included relatively accurate modeling of the antigen subunit. This suggests that most of the failed predictions can be primarily attributed to incorrect docking poses or local structural perturbations rather than inaccurate antigen subunit predictions.

Accurate subunit modeling and antibody-antigen prediction success

For comparison of predictive success with bound component inputs, we employed ZDOCK[4] with IRAD[5] scoring to perform global rigid-body docking and ranking with the bound antibody and antigen structures (with randomized initial orientations). This approach led to a relatively high proportion (62%) of cases having top-ranked predictions with medium or higher accuracy (**Fig. S14a**), indicating that both traditional docking as well as AlphaFold can successfully assemble over half, antibody-antigen complex structures with bound components, while still unable to assemble a sizable fraction of them.

Compared to using all experimentally determined chains as templates, utilizing only certain bound chains as templates resulted in decreased model accuracy. Specifically, with default antigen templates, and experimentally determined antibody heavy and light chains provided as templates, 36% of top-ranked predictions were of medium or higher accuracy (**Fig. 5c**). A similar near-native success rate (35%) was observed when using only bound antibody heavy chains as templates (**Fig. S14b**), while light chain and antigen bound chain templates led to 28% of cases with near-native top-ranked predictions (**Fig. S14c**). Overall, the resulting success rates of these bound chain template scenarios are higher than those obtained using the default templates, in which case 18% of the complexes have medium or higher accuracy top-ranked predictions. While not reflective of actual predictive modeling scenarios due to the use of bound structure information, these results indicate the potential impact of accurate and bound-

like subunit modeling, as well as its theoretical maximal effect, for AlphaFold antibody-antigen modeling success.

Inspired by the findings, we tested using modeled subunit structures as templates in antibody-antigen modeling, as we hypothesized that if these models are more accurate than the default pipeline's selected template structures, they could potentially improve AlphaFold's performance over the default pipeline and templates. However, this yielded lower success than observed when using default templates (12%, versus 18% for near-native modeling success) (**Fig. S14d**), showing that inaccuracies or deviations from the bound components for the unbound models led to far different behavior than when using actual bound components.

Utilizing antigen structures bound to other antibodies as templates

Given the practical limitations in obtaining antigens bound to the target antibodies as modeling templates, we evaluated the impact of using antigens that are bound to distinct antibodies as templates. The criteria for selecting such antigens are outlined in the Supplementary Methods. In the subset of 100 antibody-antigen complexes, we conducted such experiment on 73 antibody-antigen complexes with qualifying antigen templates identified. Results indicated that use of alternative antigen templates, compared to the original antigen template, decreased modeling success. Percentage of cases with top-ranked medium or higher accuracy predictions were 49% using bound antibody and bound original antigen (**Fig. S15a**), 41% using bound antibody and alternative bound antigen (**Fig. S15a**), 18% using default AlphaFold antibody templates and bound original antigen (**Fig. S15b**), 14% with default antibody and alternative bound antigen (**Fig. S15b**). When models were generated with bound antibody templates, the use of alternative bound antigens resulted in higher modeling success (41% cases with medium or higher top ranked predictions, **Fig. S15a**) compared to the default AlphaFold antigen templates (35%, **Fig.**

S15a). Conversely, when default AlphaFold antibody templates were employed, the success rate with alternative bound antigens (14%, **Fig. S15b**) was comparable to that achieved with default AlphaFold antigen templates (15%, **Fig. S15b**). This suggest that while alternative bound antigens can enhance model accuracy when paired with their corresponding bound antibodies, their advantage diminishes when used with generic antibody templates. Taken together, these results highlight the importance of the antibody template choice in modeling outcomes, and more broadly, deviations in modeling template from bound form led to decrease in modeling success.

Sustained v2.3 modeling success despite fewer recycles

Compared to v.2.2, v.2.3 predictions were produced with a higher number of recycles, with 95% of v.2.3 predictions generated using more than three recycles. To investigate the impact of recycles, we reduced the maximum number of recycles in v.2.3 from 20 to 5 (**Fig. S17a**) or 3 (**Fig. S17b**). Compared to the default setting with a maximum number of recycles of 20, the antibody-antigen complex prediction success remained identical for generating near-native (medium or higher accuracy) top-ranked predictions, regardless of the recycle limit.

Supplementary Figures

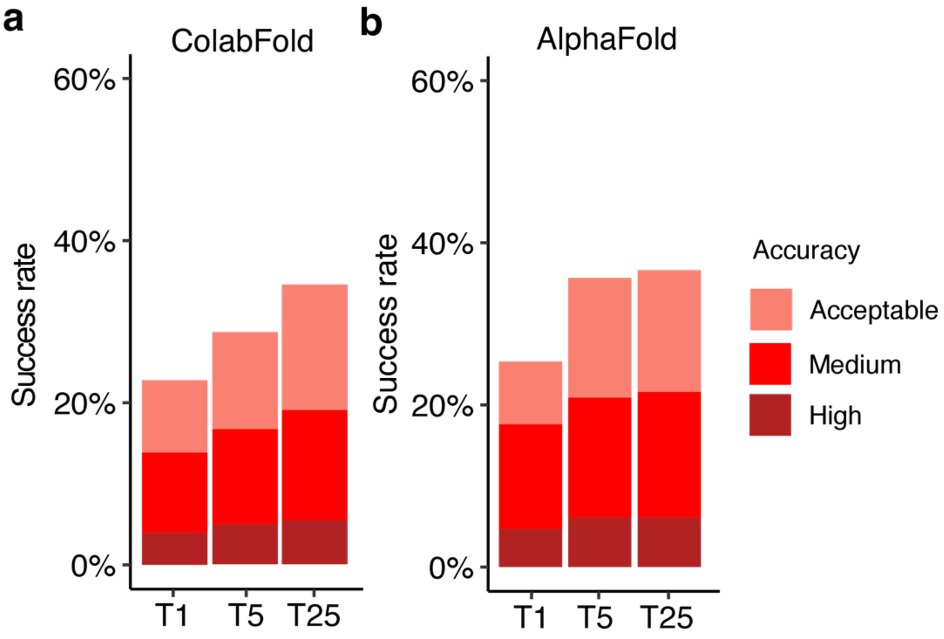

Figure S1. Antibody-antigen modeling success by ColabFold and AlphaFold. Antibody-antigen modeling success comparison of **a** ColabFold and **b** AlphaFold on 426 antibody-antigen complexes for which both algorithms successfully generated predictions. Structures released on or before April 30, 2018, were allowed as templates during modeling. For each complex, 25 predictions were generated, and were ranked by AlphaFold model confidence score. Antibody-antigen predictions were evaluated for complex modeling accuracy using CAPRI criteria for high, medium, and acceptable accuracy. The success rate was calculated based on the percentage of cases that had at least one model among their top N predictions that met a specified level of CAPRI accuracy. Bars are colored by CAPRI accuracy criteria.

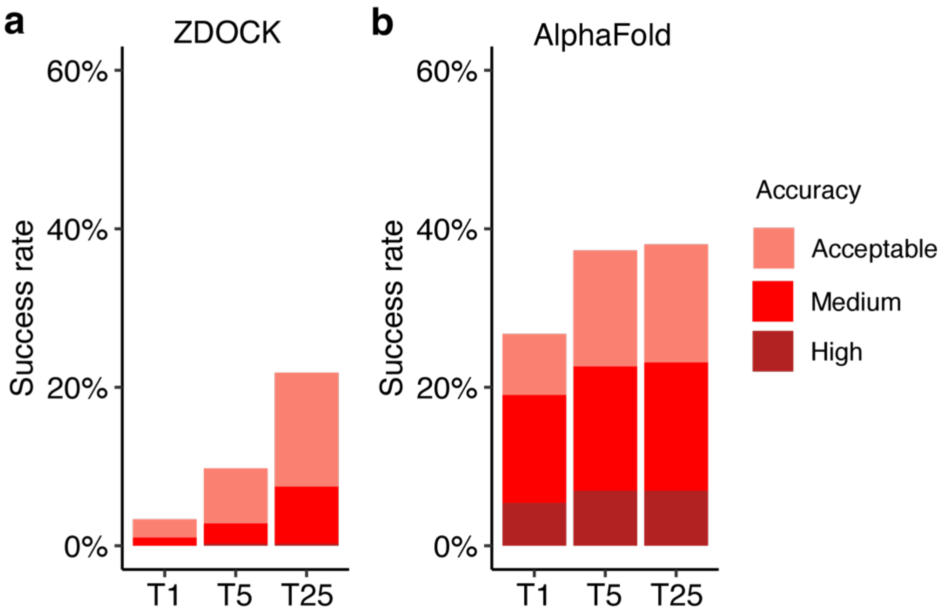

Figure S2. Antibody-antigen modeling success by ZDOCK and AlphaFold. Antibody-antigen modeling success comparison of **a** ZDOCK (version 3.0.2)[4] **b** AlphaFold on 389 antibody-antigen complexes. AlphaFold was used to generate unbound antibody and antigen structure inputs for ZDOCK. Templates released on or before April 30, 2018, were allowed during modeling. Only the top-ranked prediction, based on AlphaFold's model confidence score, was used as input. ZDOCK docking was performed only on inputs with a minimum antibody or antigen pLDDT score above 80 to ensure input model quality. ZDOCK dense sampling was employed, generating in 54,000 predictions per complex, which were subsequently ranked using IRAD[5] scores. Antibody-antigen predictions were evaluated for complex modeling accuracy using CAPRI criteria for high, medium, and acceptable accuracy. For each complex, 25 predictions were generated, and were ranked by AlphaFold model confidence score. The success rate was calculated based on the percentage of cases that had at least one model among their top N predictions that met a specified level of CAPRI accuracy. Bars are colored by CAPRI accuracy criteria.

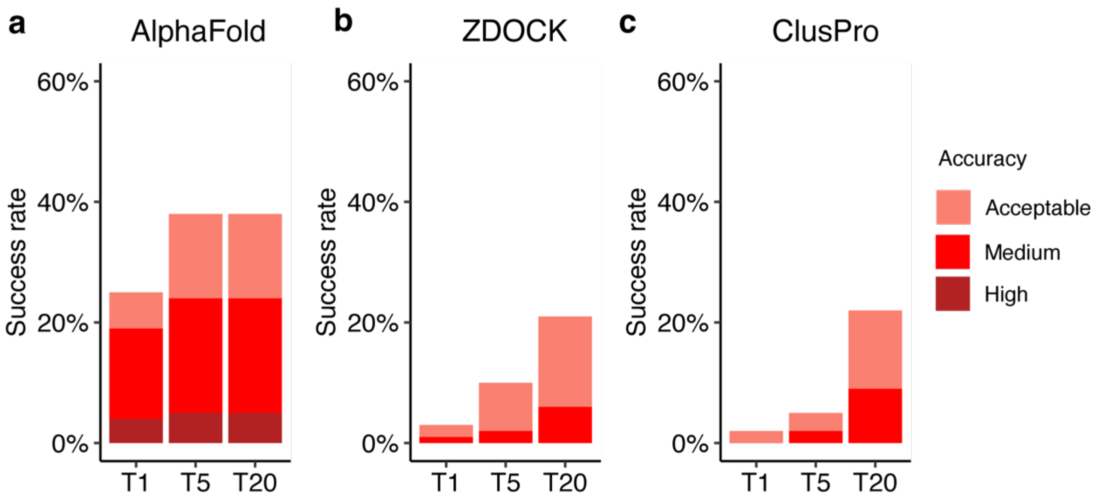

Figure S3. Antibody-antigen modeling success by AlphaFold, ZDOCK and ClusPro. Antibody-antigen modeling success comparison of **a** AlphaFold **b** ZDOCK (version 3.0.2)[4] and **c** ClusPro (antibody mode)[7] on 100 antibody-antigen complexes. For each complex, 25 predictions were generated by AlphaFold, and were ranked by AlphaFold model confidence score. AlphaFold was also used to generate unbound antibody and antigen structure inputs for ZDOCK and ClusPro. Templates released on or before April 30, 2018, were allowed during modeling. Only the top-ranked prediction, based on AlphaFold's model confidence score, was used as input. To ensure input quality, only antibody or antigen predictions with minimum pLDDT score above 80 were used. ZDOCK dense sampling was employed, generating in 54,000 predictions per complex, which were subsequently ranked using IRAD[5] scores. ClusPro antibody mode and automatic masking of non-CDR regions were enabled. Modeling success up to top 20 predictions were shown in the figure, as ClusPro generated 20-30 predictions for most cases, except for case 7aqy, where ClusPro produced only 14 predictions. Antibody-antigen predictions were evaluated for complex modeling accuracy using CAPRI criteria for high, medium, and acceptable accuracy. The success rate was calculated based on the percentage of cases that had at least one model among their top N predictions that met a specified level of CAPRI accuracy. Bars are colored by CAPRI accuracy criteria.

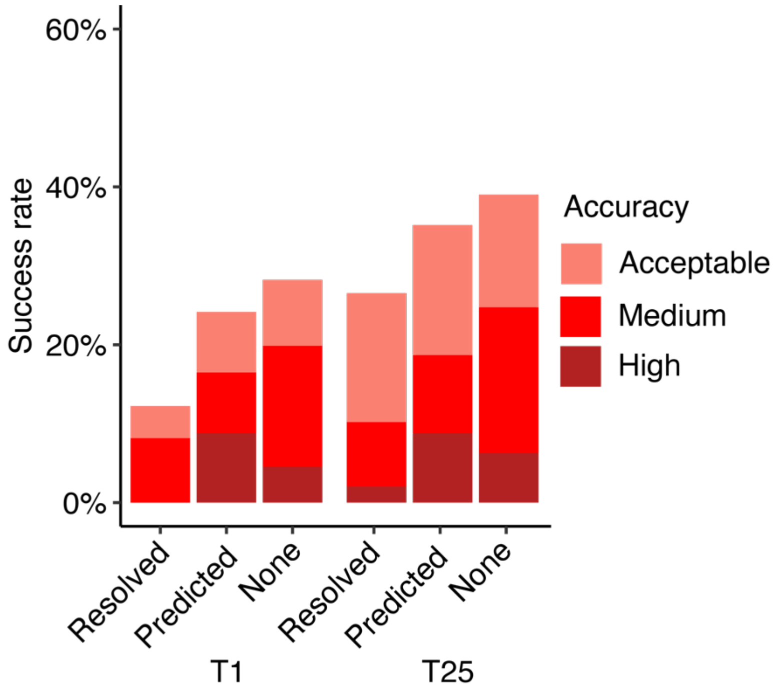

Figure S4. Presence of experimentally resolved and predicted interface glycans on modeling success. Complexes were classified as either "Resolved" (glycans or ligands found in antibody-antigen chain interface in experimentally resolved structures, N=49), "Predicted" (glycans not found in antibody-antigen interface of experimentally resolved structures but the antibody-antigen complex was predicted to have antibody-proximal antigen glycosylation, N=91), and "None" (none of the above, N=287). Bars were colored by CAPRI criteria.

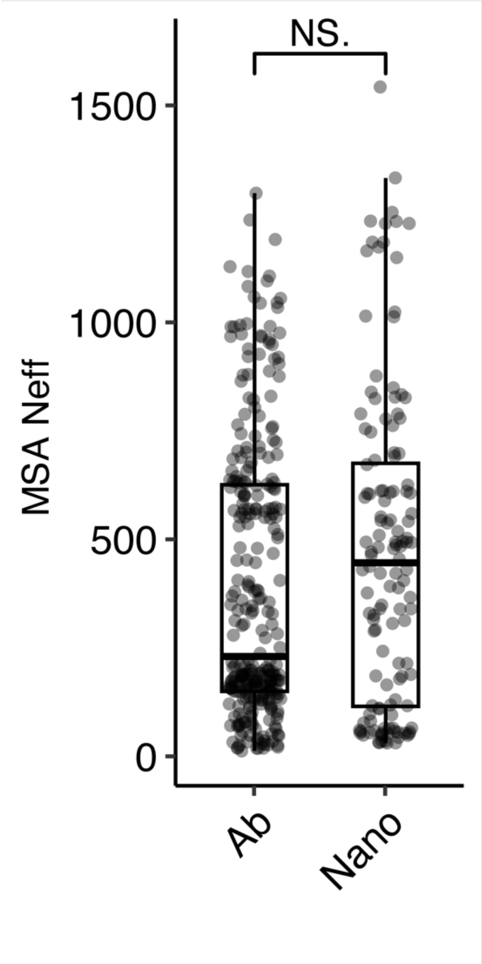

Figure S5. Distribution of MSA depth (Neff) grouped by antibody type. Based on the antibody type, complexes are categorized into heavy-light chain antibody-antigen complexes (Ab, N=294), or nanobody/VHH-antibody complexes (Nano, N=132). Statistical significance values (Wilcoxon rank-sum test) were calculated between MSA depth for antibody targets versus nanobody targets, as noted at top (NS.: not significant, $p > 0.05$).

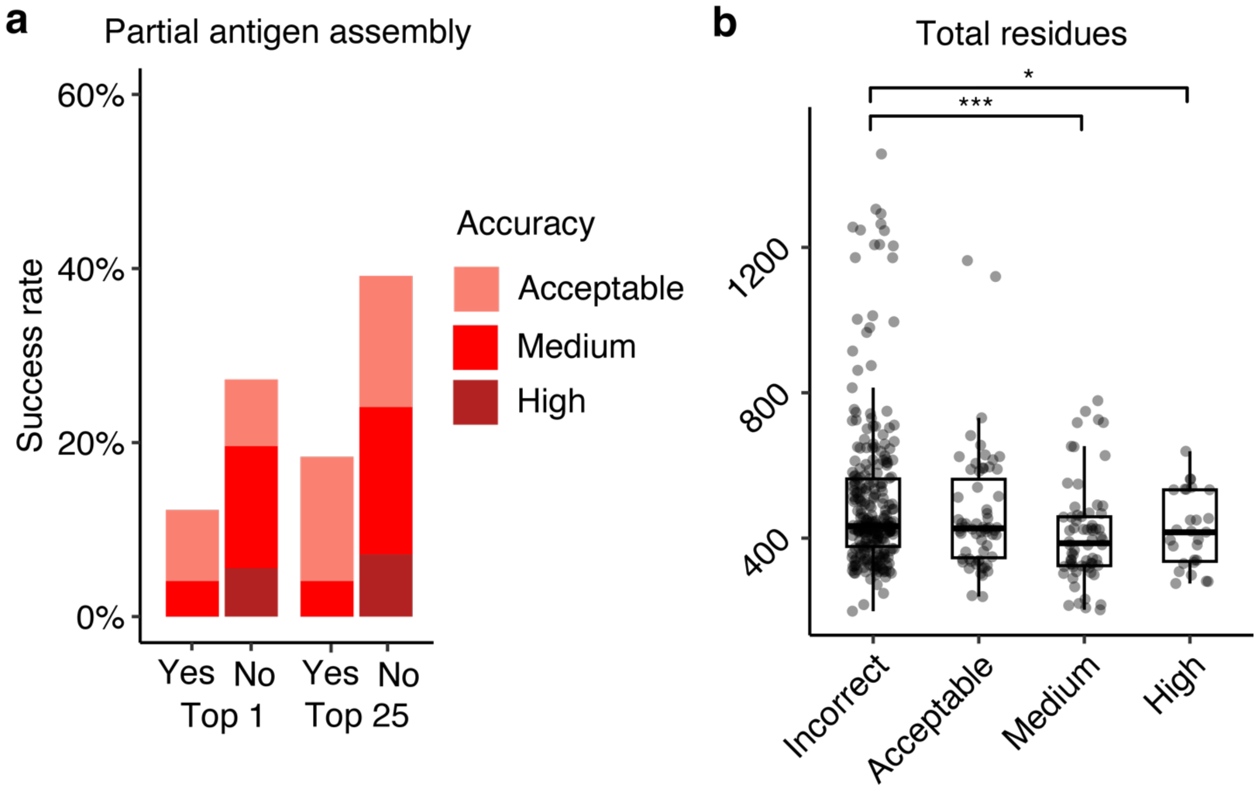

Figure S6. Antibody-antigen modeling success determinants. A Partial versus full antigen assembly input. Complexes were classified as either "Yes" (N=49) or "No" (N=378) to indicate whether a partial antigen assembly was modeled, meaning that the antigen was modeled without additional chains that are present in the full PDB bioassembly. T1 and T25 denote AlphaFold modeling accuracy in top 1 (ranked by AlphaFold model confidence score) and in all 25 predictions of the complex. Bars are colored by CAPRI criteria. **b** Total number of residues in the complex grouped by AlphaFold modeling success. The modeling success is defined as the highest CAPRI criteria prediction in the complex, considering all 25 predictions. Statistical significance values (Wilcoxon rank-sum test) were calculated between total residue counts for sets of cases with incorrect versus medium and incorrect versus high CAPRI accuracy predictions, as noted at top (*$p \leq 0.05$, ***$p \leq 0.001$).

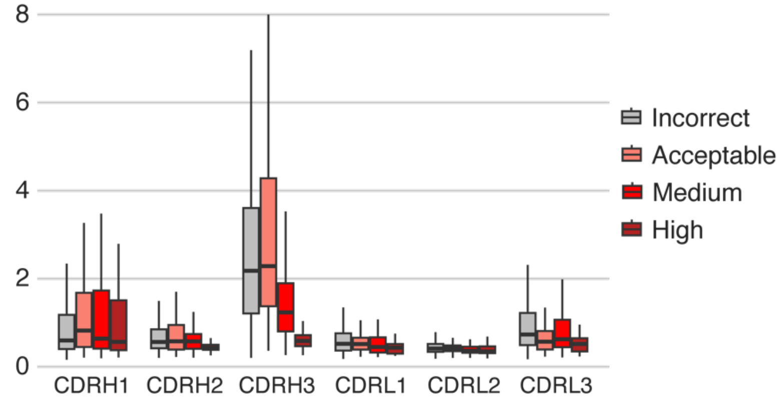

Figure S7. Distribution of CDR modeling accuracy, grouped by CDR type and by AlphaFold modeling accuracy. AlphaFold modeling accuracy is defined as the accuracy of highest CAPRI criteria prediction in the complex, considering all 25 predictions. For CDRH1, CDRH2 and CDRH3, the numbers of data points in each category are 261, 60, 62, 25 for incorrect, acceptable, medium and high success groups. For CDRL1, CDRL2 and CDRL3, the numbers of data points in each category are 192, 45, 39, 10 for incorrect, acceptable, medium, and high success groups. Bars are colored by CAPRI model accuracy.

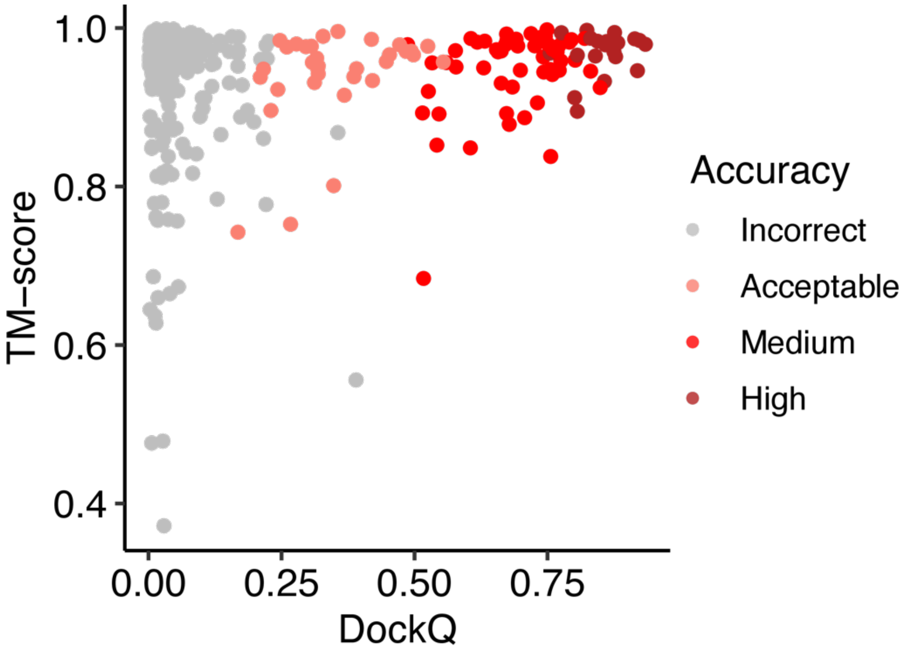

Figure S8. Relationship between antigen modeling accuracy and complex prediction accuracy. Top-ranked predictions of 427 complexes generated by AlphaFold are represented as data points. The antigen accuracy is measured by TM-score of the top-ranked prediction, and the complex model accuracy measured by DockQ score. If the antigen has multiple chains, the minimum TM-score of all antigen chains is selected as the antigen TM-score. Data points are colored by CAPRI model accuracy.

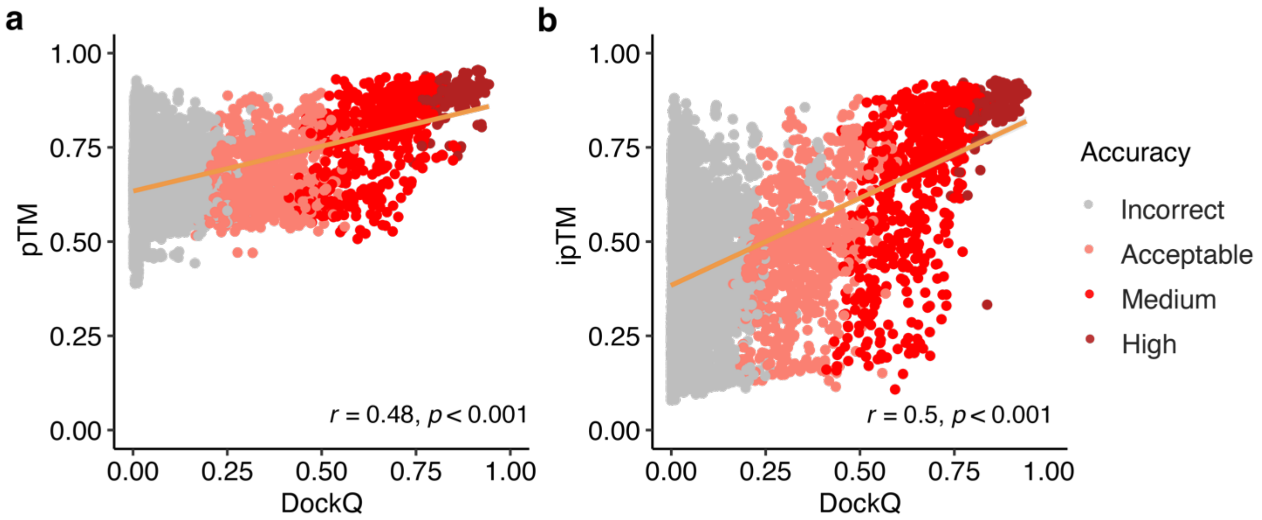

Figure S9. **Relationship between model confidence scores and model accuracy.** Scatter plots depicting the association between the **a** pTM, **b** ipTM scores and DockQ scores. In the scatter plots, all 25 models representing 427 complexes are depicted as data points, with their colors indicating the model quality according to CAPRI criteria. The orange line represents the linear regression, and the lower right corner of the scatter plots displays the Pearson's correlation coefficients and correlation p-values.

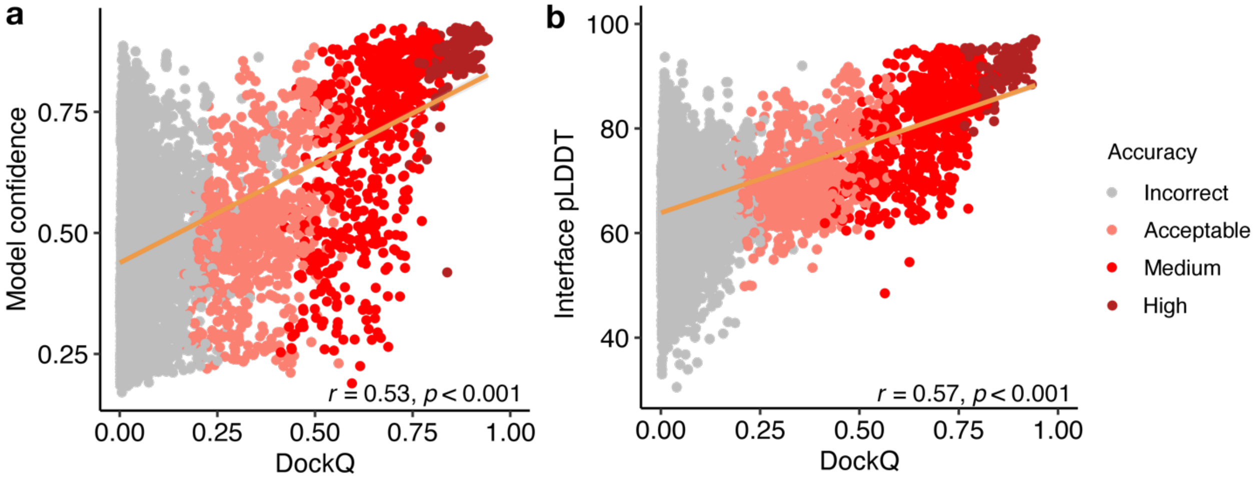

Figure S10. **Relationship between model scores and model accuracy.** Scatter plots depicting the association between the **a** model confidence, **b** interface pLDDT scores and the DockQ scores. A total of 10,236 data points were present in each scatter plot, which include all 25 models representing 427 complexes, excluding models without side-chain contacts within 4 Å across the antibody-antigen interface. Data points are colors indicating the model quality according to CAPRI criteria. The orange line represents the linear regression, and the lower right corner of the scatter plots displays the Pearson's correlation coefficients and correlation p-values.

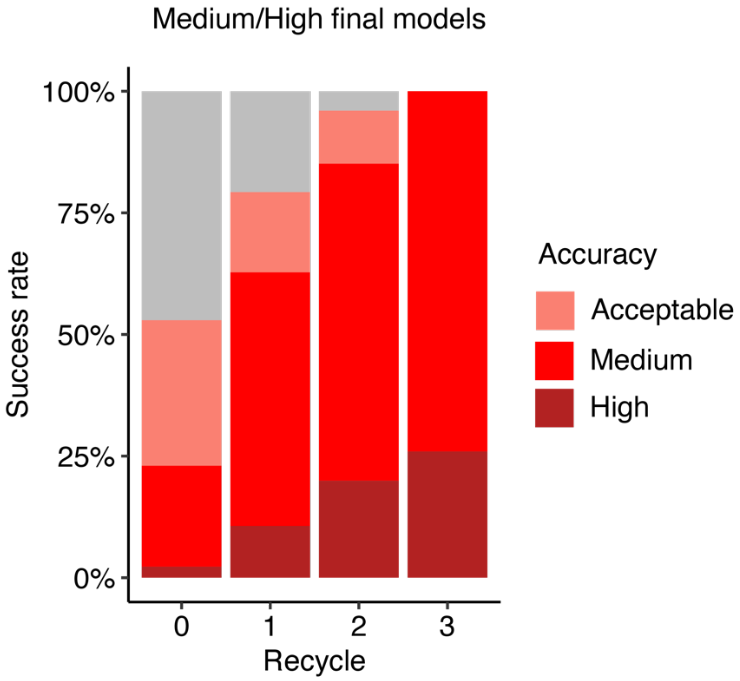

Figure S11. Analysis of antibody-antigen predictions accuracy across recycling iterations. The modeling success of complexes at each recycle focusing on a subset of predictions that reached medium or higher accuracy after 3 recycling iterations (N=106). Recycle=0 denotes the state of the prediction before recycling iterations begin.

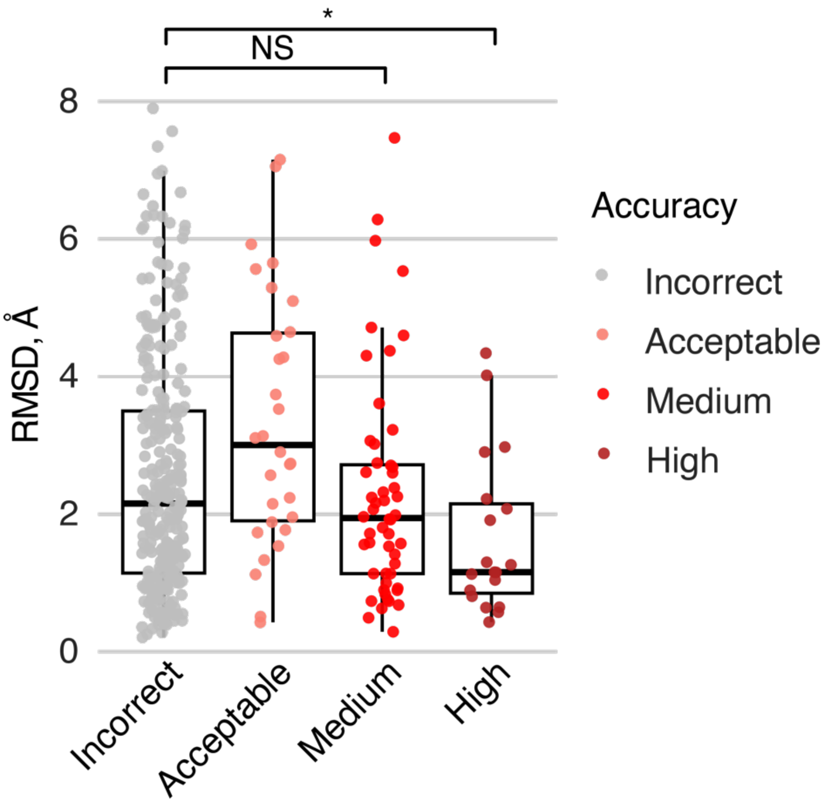

Figure S12. The distribution of CDRH3 modeling accuracy in top-ranked unbound antibody model grouped by top-ranked complex modeling success. The modeling success is CAPRI criteria of top-ranked complex prediction generated by AlphaFold. The CDRH3 RMSD measures the RMSD of the top-ranked unbound antibody prediction generated by AlphaFold. Numbers of data points in incorrect, acceptable, medium, and high categories are 304, 31, 52, 19. Statistical significance values (Wilcoxon rank-sum test) were calculated between RMSD values for sets of cases with incorrect versus medium and incorrect versus high CAPRI accuracy predictions, as noted at top (NS: $p > 0.05$, *$p \leq 0.05$).

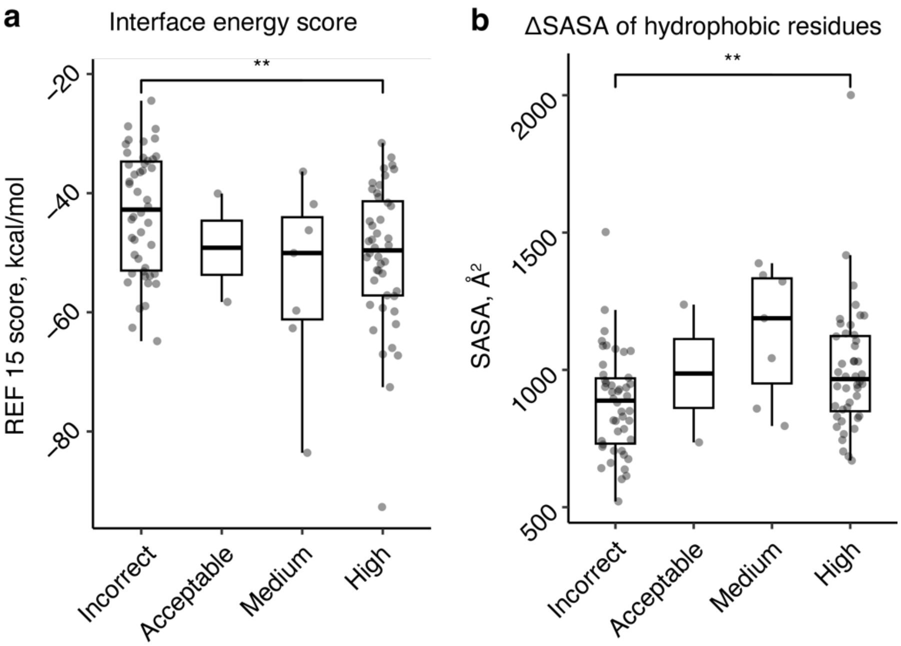

Figure S13. Antibody-antigen modeling success determinants of AlphaFold with bound antibody and antigen structures as templates. Distribution of **a** interface energy score and **b** change in solvent-accessible surface area (ΔSASA) of hydrophobic part of the antibody-antigen interface (calculated by the Rosetta InterfaceAnalyzer program), with cases grouped by complex modeling success for AlphaFold when using bound antibody and antigen structures as templates. The modeling success is defined as the highest CAPRI criteria prediction in the complex, considering all 5 predictions. Numbers of data points in incorrect, acceptable, medium and high categories are 46, 2, 7 and 45. Statistical significance values (Wilcoxon rank-sum test) were calculated between scores for sets of cases with incorrect versus high CAPRI accuracy predictions, as noted at top (**$p \leq 0.01$).

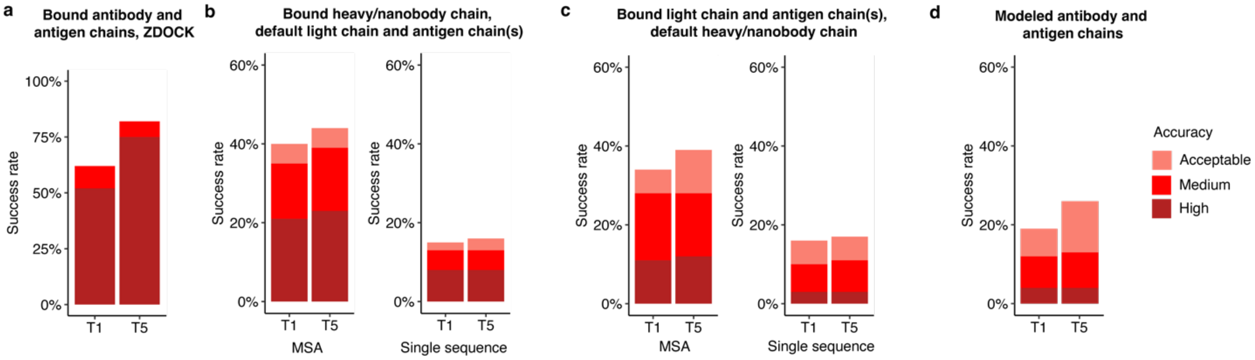

Figure S14. Antibody-antigen modeling success using varying template inputs. Antibody-antigen modeling success of **a** ZDOCK (version 3.0.2) with IRAD re-ranking of dense-sampling predictions (54,000 predictions per complex), utilizing bound antibody and bound antigen chains as docking input, and of AlphaFold by utilizing **b** bound heavy/nanobody chain, and default light chain and antigen chains as templates, **c** bound light chain, and default heavy/nanobody chain and antigen chains as templates, **d** antibody and antigen chains modeled by AlphaFold as templates. A template date cutoff of 2018-04-30 was applied to identify default templates. Benchmarking was performed on a total of 100 antibody-antigen complexes. The success rate was calculated based on the percentage of cases that had at least one model among their top N predictions that met a specified level of CAPRI accuracy. Bars are colored by CAPRI accuracy criteria.

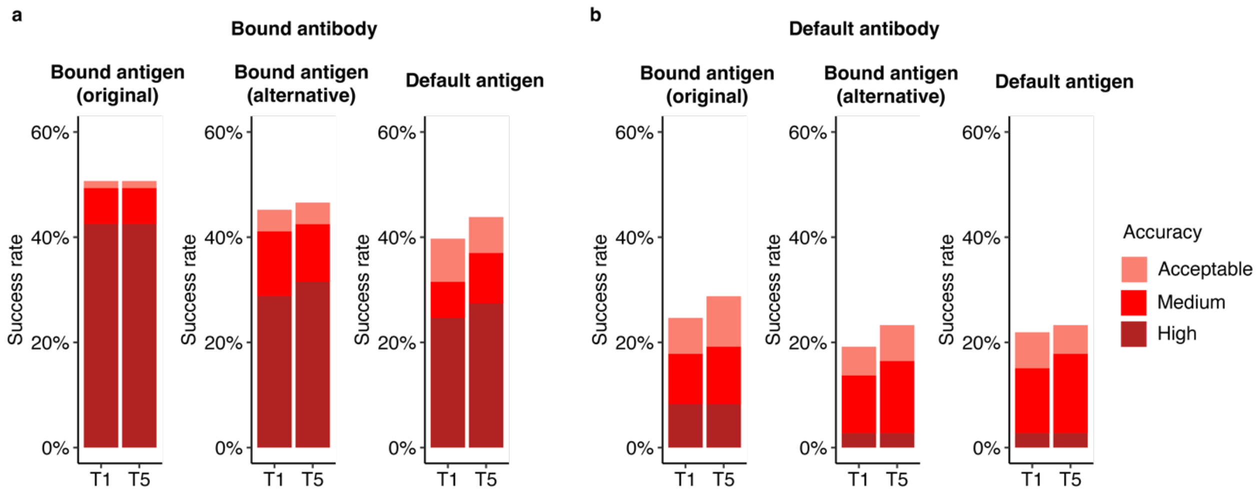

Figure S15. Antibody-antigen modeling success using antigens bound to alternative antibodies as template inputs. AlphaFold generated antibody-antigen complexes using **a** bound antibody and **b** default antibody, coupled with bound antigen (original), bound antigen (alternative) and default antigen as templates. A template date cutoff of 2018-04-30 was applied to identify default templates. Benchmarking was performed on a total of 73 antibody-antigen complexes. The success rate was calculated based on the percentage of cases that had at least one model among their top N predictions that met a specified level of CAPRI accuracy. Bars were colored by CAPRI accuracy criteria.

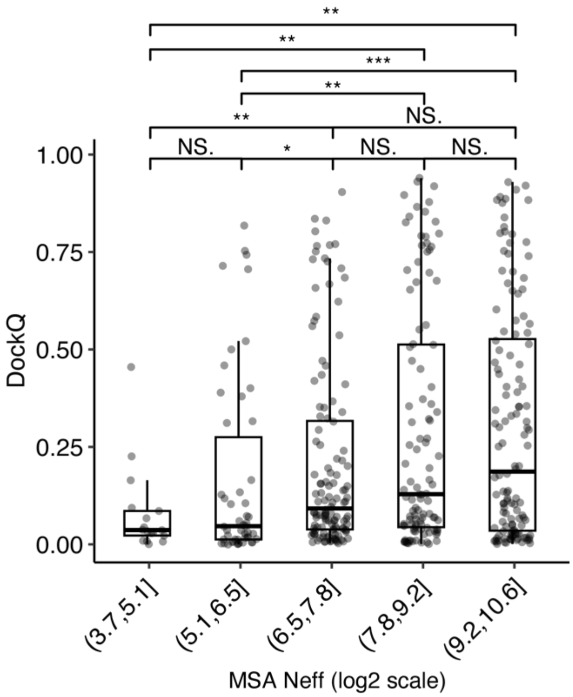

Figure S16. Distribution of DockQ scores grouped by ranges of AlphaFold MSA depth. The DockQ scores selected for each individual data points are the highest DockQ score of all 25 complex predictions generated by AlphaFold. Numbers of data points in (3.69,5.08], (5.08,6.46], (6.46,7.84], (7.84,9.21] and (9.21,10.6] Neff ranges are 17, 50, 124, 107, 128 respectively. Statistical significance values (Wilcoxon rank-sum test) were calculated between DockQ scores for sets of cases with varying ranges of MSA depth, as noted at top (NS.: $p > 0.05$, *$p \leq 0.005$, **$p \leq 0 .001$, ***$p \leq 0.001$).

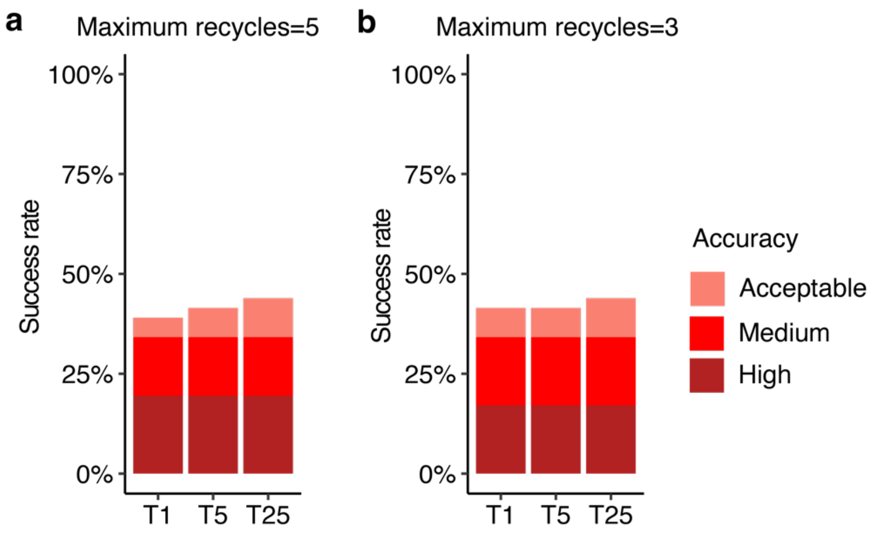

Figure S17. Antibody-antigen modeling success comparison of AlphaFold (v.2.3) using a varying number of maximum recycles. The maximum number of recycling iterations were set to **a** 5 and **b** 3, and tested on 41 antibody-antigen complexes. Templates released on or before September 30, 2021, were allowed during modeling. For each complex, 25 predictions were generated, and were ranked by AlphaFold model confidence score. Antibody-antigen predictions were evaluated for complex modeling accuracy using CAPRI criteria for high, medium and acceptable accuracy. The success rate was calculated based on the percentage of cases that had at least one model among their top N predictions that met a specified level of CAPRI accuracy. Bars were colored by CAPRI accuracy criteria.

Supplementary Table

Table S1. Antibody-antigen structures used for AlphaFold benchmarking.

PDB	Heavy chain	Light chain	Antigen chain(s)	Release Date	v.2.3.0 set[1]	100 subset[2]
6was	H	L	J	3/31/21		X
6p50	H	L	C	9/4/19		
6urm	H	L	F	9/16/20		
6umg	h	l	cr	2/12/20		
6a0z	H	L	A	6/20/18		X
7daa	H	L	A	10/20/21	X	X
6s8j	P	O	C	2/12/20		
6s8i	P	O	C	2/12/20		
7lf7	A	B	M	8/4/21		
6urh	H	L	C	3/18/20		
7lfb	H	L	X	8/4/21		
6ktr	A	B	C	7/8/20		
6meh	H	L	C	11/21/18		
6vel	H	L	C	1/29/20		X
6y9b	I	M	A	5/20/20		
6xkq	H	L	A	10/14/20		
6o9h	H	L	D	1/22/20		
6yio	H	L	B	11/11/20		
7l7r	B	A	G	12/1/21	X	
7l7r	D	C	G	12/1/21	X	X
6svl	A	B	C	11/27/19		
6okm	H	L	R	8/28/19		
6z3p	H	L	CAB	9/2/20		
6u36	H	L	B	11/6/19		
6jbt	H	L	F	6/19/19		
6wbv	H	L	A	9/9/20		
7jmp	H	L	A	8/26/20		X
6wo5	G	I	F	8/19/20		X
7lfa	B	D	A	8/4/21		
7np1	H	L	A	11/17/21		
6hig	H	L	B	6/5/19		
6vyh	D	C	A	11/11/20		
6k65	H	L	A	8/14/19		
6umx	h	l	B	2/26/20		
7nx3	B	C	F	10/27/21	X	

7jv6	C	D	B	10/14/20	
6xqw	H	L	E	3/3/21	
7jtg	A	B	E	3/10/21	X
6o9i	D	E	C	1/22/20	X
7e7x	H	L	A	6/9/21	
6wo5	A	B	E	8/19/20	
6mvl	H	L	A	10/23/19	
7kqg	B	C	A	12/16/20	
6glw	H	L	A	6/5/19	
6gku	H	L	A	6/5/19	
7lue	H	L	A	6/16/21	
7k9j	J	N	C	9/29/21	
7n8h	C	B	A	7/14/21	
6wo3	H	L	E	8/19/20	
6wmw	M	N	B	7/15/20	
6wmw	H	L	B	7/15/20	
7czx	I	M	B	3/10/21	
7czw	H	L	A	3/10/21	
7rah	B	A	E	9/15/21	
7rah	D	C	E	9/15/21	
7kn4	M	N	B	9/22/21	
6nyq	H	L	C	1/22/20	
6xkr	H	L	P	9/9/20	X
6jjp	D	E	F	10/30/19	
7lxy	N	O	J	4/14/21	
7lr4	H	L	D	12/15/21	
7r89	C	D	BA	9/8/21	
6ywc	D	E	F	10/7/20	
6xxv	D	E	F	4/22/20	
7ly2	N	O	J	4/14/21	
6vvu	G	I	D	12/30/20	
6k0y	A	B	C	12/11/19	X
6v4o	H	L	N	10/7/20	
6wgl	A	B	C	9/16/20	
6xsw	D	E	F	7/21/21	
6hx4	H	L	B	10/30/19	
6wzk	A	B	E	11/25/20	
7lxz	H	J	A	4/14/21	
7ceb	C	D	B	6/23/21	
6lxi	C	D	B	12/2/20	

6osv	H	L	K	4/1/20		
7lbg	H	G	A	3/10/21		
7lbg	F	E	A	3/10/21		X
6a3w	A	B	C	10/10/18		
7bnv	H	L	A	11/17/21	X	X
7lm9	H	L	A	3/31/21		
7o52	H	L	U	7/28/21		
7s4s	H	L	A	9/22/21		
7lop	X	Y	Z	3/3/21		
6xkp	M	N	B	10/14/20		
6h2y	H	L	D	8/14/19		
6phc	C	D	E	10/2/19		X
6osh	H	L	K	4/1/20		
7n3d	H	L	C	7/7/21		
7e7y	A	B	R	6/9/21		
7chf	A	B	R	9/16/20		X
7lm8	H	L	A	3/31/21		X
7kn3	M	N	B	9/22/21		X
7n0u	H	L	C	8/11/21		
7joo	H	L	C	10/14/20		
7dk2	D	E	F	12/8/21		X
6sni	H	L	X	3/11/20		
7c88	A	B	C	4/14/21		X
7dc8	B	A	C	1/13/21		
7mmo	D	E	F	5/12/21		
7kfv	F	G	E	12/2/20		X
7k9z	B	A	E	10/28/20		
6mlk	H	L	A	10/17/18		
6mg7	H	L	G	9/25/19		X
7l0l	H	L	BA	11/3/21	X	
6ogx	C	D	G	7/10/19		
7lcv	A	B	C	6/9/21		
6pi7	F	E	D	7/24/19		
7ps6	H	L	E	12/15/21	X	X
6rlo	G	H	L	5/12/21		
7lab	Y	X	B	3/10/21		
7cm4	H	L	A	1/20/21		
6gv4	H	L	BA	11/21/18		
6j14	A	B	G	11/6/19		
6ohg	C	B	A	6/17/20		

6v4n	D	E	W	10/7/20		
7e5o	H	L	A	9/8/21		X
6p67	A	B	K	9/4/19		
7ean	H	L	A	3/31/21		X
7eam	H	L	A	3/17/21		
6ppg	B	A	G	12/11/19		X
6nmr	J	K	M	8/7/19		
7djz	A	B	C	6/9/21		X
6m3b	C	B	A	7/8/20		X
6o1f	H	L	AI	10/16/19		
7lr3	H	L	D	12/15/21		
6r8x	C	B	A	4/10/19		X
6wxl	F	E	DC	6/9/21		
6a67	H	L	A	8/29/18		
6mej	H	L	C	11/21/18		X
6mej	A	B	C	11/21/18		
6udj	H	I	J	1/29/20		
7cho	B	C	A	5/19/21		X
7bwj	H	L	E	6/3/20		X
7kqb	H	L	A	5/26/21		
6kz0	K	L	J	5/27/20		
7vux	H	L	A	11/17/21	X	
7q0i	H	L	C	12/22/21	X	X
7orb	E	F	X	7/7/21		
6nmu	B	A	C	8/7/19		
7s0b	C	D	E	10/6/21		
6rps	H	L	A	11/13/19		
7l7e	O	P	K	9/1/21		
7orb	H	L	R	7/7/21		
7r6w	A	B	R	7/21/21		
7r6w	H	L	R	7/21/21		
7kyl	H	L	E	4/7/21		
6iut	H	L	A	1/16/19		
6nmt	B	A	C	8/7/19		
6dkj	A	B	D	5/8/19		X
7c61	H	L	A	7/29/20		
7m3n	H	L	A	7/28/21		
7s13	H	L	D	10/20/21		
7m7w	C	D	S	5/5/21		
6nmv	H	L	S	8/7/19		X

6icc	H	L	A	2/13/19		
7m7w	H	L	R	5/5/21		X
6j15	A	B	C	11/6/19		X
6whk	C	B	A	4/14/21		
7kpb	H	L	AC	1/13/21		
6pe8	H	L	T	8/14/19		
7ket	A	B	C	6/9/21		
6ion	H	L	A	1/15/20		X
6nms	B	A	C	8/7/19		
6lz9	H	L	B	3/11/20		X
6p3r	C	D	E	5/27/20		
7coe	B	C	D	8/4/21		
7ps4	H	L	E	12/15/21	X	
7ps1	A	B	E	12/15/21		X
6phb	D	C	I	10/2/19		
6lgw	C	D	F	2/19/20		
7s11	I	M	D	11/3/21	X	
6ieb	E	F	B	4/10/19		
6e63	H	L	P	11/28/18		X
7seg	H	L	C	11/24/21		X
6wh9	E	F	D	9/9/20		
7ps0	H	L	E	12/15/21	X	X
6xlq	B	C	A	9/2/20		
7dm1	D	C	A	12/23/20		
7dm2	H	L	A	12/23/20		X
6j5d	H	L	A	2/6/19		
7o9s	H	L	A	6/23/21		X
7kmg	D	E	F	1/27/21		
6q0e	H	L	A	12/18/19		
7cr5	H	L	A	3/24/21		
7q0g	A	B	E	12/22/21	X	X
6h3t	I	M	B	2/27/19		
6s5a	H	L	DA	9/25/19		
7dha	C	B	A	9/22/21		
6ddm	B	A	C	10/24/18		
7mzm	H	L	A	10/6/21	X	X
6nha	H	L	AB	12/25/19		
6xpx	B	C	A	5/19/21		
6xq0	E	F	D	5/19/21		
6e56	H	J	A	5/22/19		

7ps2	H	L	G	12/15/21	X	X
7ps2	A	B	G	12/15/21		X
6tyb	H	L	G	10/2/19		
6u9s	D	E	F	5/13/20		X
7bq5	H	L	A	3/24/21		
7dr4	A	B	J	4/14/21		X
6m58	C	D	B	4/29/20		
6ss2	H	L	A	6/10/20		
7kzb	H	L	C	2/3/21		X
6vug	D	C	B	2/17/21		
6e4x	Z	Y	B	5/22/19		X
6otc	H	L	A	6/5/19		
6oy4	D	C	A	8/28/19		X
7nx8	H	L	E	4/7/21		X
7bel	C	D	X	3/3/21		
7mzg	H	L	A	10/6/21		
7bek	H	L	E	3/3/21		
7mf1	H	L	A	5/12/21		
7bei	H	L	E	3/3/21		X
7bel	E	F	X	3/3/21		
6iuv	C	D	B	1/16/19		X
6iea	H	L	A	4/10/19		
7e3o	H	L	R	9/15/21		
7ahu	B	A	EF	7/7/21		
6q18	H	L	A	12/18/19		
7kmh	A	B	C	1/27/21		
7mzk	N	M	B	10/6/21	X	
7or9	H	L	E	7/7/21		X
6oz2	H	L	G	8/19/20		
6xzw	H	L	D	10/14/20		X
6iek	B	C	A	4/10/19		X
7rks	I	M	S	9/22/21		
6w7s	H	L	A	9/9/20		X
7mzi	H	L	A	10/6/21		
7neh	H	L	E	3/3/21		
7mzj	H	L	A	10/6/21	X	X
6i8s	E	I	A	2/13/19		
6oor	H	L	A	7/17/19		
6vy6	H	L	A	1/6/21		X
7bbj	H	L	A	12/29/21	X	

6mfp	C	D	A	9/18/19		
6wm9	E	F	D	1/27/21		
7mzh	H	L	E	10/6/21		
7bep	A	B	E	3/3/21		
7chz	H	L	I	1/13/21		X
6qig	H	L	A	9/4/19		
6hhc	H	L	A	9/11/19		X
6woz	K	L	J	1/27/21		
7lsg	H	L	C	4/7/21		X
7jx3	C	D	R	10/14/20		
7jx3	H	L	R	10/14/20		X
6j6y	E	F	D	8/7/19		
7kq7	H	L	B	4/7/21		
6ztr	A	B	J	5/5/21		X
7ce2	Z	B	A	4/7/21		
6wtu	E	F	D	1/27/21		
6ocb	H	L	A	5/29/19		
6wds	H	L	CAB	7/15/20		
7kyo	H	L	B	8/25/21		
6o39	B	A	C	4/3/19		
6ba5	F	E	O	6/13/18		
6n6b	K	L	A	7/3/19		
6ye3	G	H	I	12/30/20		X
7bsc	H	L	A	12/23/20		
6kyz	B	C	A	5/27/20		
7ec5	E	F	BAC	3/31/21		
6mhr	A	B	C	11/21/18		
6pzf	F	E	B	12/4/19		
6pze	H	L	A	12/4/19		
6z2l	C	B	A	7/22/20		
6e3h	H	L	BA	9/26/18		
6cxy	H	L	C	4/10/19		
6nz7	H	L	BA	5/8/19		
6vy4	C	D	B	12/30/20		
7e72	C	D	F	11/10/21	X	X
7n4j	H	L	A	10/6/21	X	
6e62	H	L	P	11/28/18		
6q20	H	L	A	10/23/19		
6vc9	H	L	A	11/11/20		
6lyn	H	L	D	2/24/21		

6ivz	H	L	A	2/13/19	
6id4	C	D	EF	2/6/19	
6hxw	C	D	B	8/28/19	
7d85	E	F	D	4/7/21	
7r8l	H	L	E	8/4/21	
7klh	H	L	A	2/10/21	
7mhy	O	P	A	6/16/21	X
7mhy	M	N	A	6/16/21	
6hga	H	L	B	3/18/20	
6pxh	H	L	B	9/25/19	
7cj2	K	L	B	7/14/21	
6j11	F	G	B	7/24/19	
7jie	E	F	A	6/30/21	
6n5e	G	F	B	6/5/19	
6u6u	H	L	R	4/22/20	
6iap	E	D	A	6/12/19	
6iap	H	L	A	6/12/19	X
7n3c	H	L	C	7/7/21	
7e9b	H	L	C	7/28/21	
7kpg	H	L	S	12/16/20	
6jep	H	L	E	5/15/19	
6dfj	H	L	E	10/24/18	X
6ute	C	D	S	4/15/20	X
6ddr	B	A	C	10/24/18	X
6ddv	B	A	C	10/24/18	X
6a77	H	L	A	1/30/19	
6rvc	D		A	10/2/19	
6gju	C		A	6/26/19	
6gjq	B		A	6/19/19	
6r7t	A		B	5/1/19	
7l1v	S		R	2/10/21	
7my3	E		A	6/16/21	
7kkl	E		C	11/11/20	
6v7y	F		A	9/16/20	
6x04	H		G	12/9/20	X
7rnn	C		D	8/11/21	
7p77	A		B	8/4/21	
6u54	A		B	11/6/19	
7mjh	F		C	5/12/21	
7my2	H		E	6/16/21	

7kn6	C	A	1/20/21		X
7kjh	A	C	2/3/21		
7o06	A	C	9/8/21		X
6zxn	E	B	9/23/20		
7a29	E	B	10/21/20		
6ze1	B	A	6/30/21		X
7d6y	B	A	10/6/21		
6rnk	B	A	8/14/19		
6v7z	F	AB	9/16/20		
7kn5	E	A	1/20/21		
7kn5	C	A	1/20/21		X
6u55	A	B	11/6/19		
6yu8	B	A	2/17/21		
7apj	B	A	8/25/21		
6x05	K	A	12/9/20		
7olz	B	A	8/11/21		
7olz	C	A	8/11/21		
6qup	B	A	8/5/20		X
6qgw	B	A	6/26/19		
6z20	D	C	9/23/20		
6rqm	B	A	7/8/20		
6oyh	E	A	7/10/19		
6os2	D	A	2/19/20		X
6qgx	B	A	6/26/19		
6qgy	B	A	6/26/19		
6z1v	B	A	9/23/20		
7o0s	A	B	9/15/21		
7r98	F	C	7/7/21		
7c8v	A	B	6/24/20		
6ir1	B	A	11/13/19		X
7cz0	E	A	9/8/21		
6x07	B	A	12/9/20		X
7nfq	C	A	12/1/21	X	
7nfr	B	A	12/1/21	X	
6lz2	B	A	12/23/20		
6gs4	H	A	1/30/19		
6gk4	F	D	6/19/19		
6o3c	B	A	7/3/19		
7nx0	D	C	10/27/21	X	
6yz5	F	E	6/3/20		

6z6v	G	BC	6/10/20		
6rtw	B	A	10/2/19		
7k84	B	A	10/14/20		
6uc6	D	B	3/4/20		
7lzp	G	A	12/22/21		X
6oq6	D	A	7/10/19		
6ir2	B	A	11/13/19		X
6gkd	B	A	6/19/19		
7d30	A	B	2/17/21		
7lzp	F	D	12/22/21		X
7lzp	E	D	12/22/21	X	
6mxt	N	A	11/14/18		
6dbg	C	B	7/18/18		
6vbg	D	B	11/25/20		
7azb	B	A	11/25/20		
6gwn	B	A	1/1/20		
6gwn	C	A	1/1/20		
6zg3	E	AI	3/3/21		
7a0v	B	A	12/30/20		
6ssi	J	E	2/12/20		
6gjs	C	A	6/26/19		
6gjs	B	A	6/26/19		
6lr7	B	A	4/29/20		X
7vnb	A	B	11/24/21	X	
7ldj	G	C	5/5/21		X
7anq	B	A	10/20/21	X	X
6h02	B	A	8/29/18		
6hhu	G	A	7/24/19		X
6hhu	H	A	7/24/19		
6uft	B	A	3/4/20		X
7e53	B	A	10/13/21	X	
6oca	C	A	4/1/20		
7m1h	G	A	12/22/21	X	
7m1h	E	A	12/22/21	X	
7m1h	F	A	12/22/21	X	
7kdu	C	BA	8/4/21		
6fyu	C	BA	11/7/18		
6h6y	G	C	12/19/18		
7na9	D	A	12/22/21	X	X
6ui1	D	A	3/4/20		X

7t5f	E	D	12/29/21	X	
7kc9	F	ED	8/4/21		
7lvw	I	D	3/24/21		
7aqy	C	B	11/3/21	X	
6zrv	B	A	8/26/20		
7t5f	C	A	12/29/21	X	X
7kbk	C	AB	8/4/21		
7kd2	C	BA	8/4/21		
6ul6	C	A	3/4/20		
6ul6	B	A	3/4/20		
6i8g	B	A	10/2/19		
6h16	B	A	1/30/19		
7kd0	C	BA	8/4/21		
7aqz	D	A	11/3/21		
7l6v	B	A	12/22/21	X	
7l6v	D	A	12/22/21	X	
7l6v	C	A	12/22/21	X	
7l6v	F	A	12/22/21	X	
6uht	C	A	3/4/20		
7n0r	D	B	6/2/21		
6oq7	C	A	7/10/19		
6rah	C	B	7/31/19		
7ar0	B	A	11/3/21	X	
6xw4	C	A	4/22/20		
6h15	D	B	1/30/19		
6h72	C	A	12/19/18		
7aqx	D	B	11/3/21		
6sge	B	A	9/25/19		
6waq	A	B	4/1/20		
7n0i	L	GH	6/9/21		
7d2z	A	B	2/17/21		
6ocd	B	A	4/1/20		
6tej	C	B	4/1/20		
6oq8	D	A	7/10/19		
6b20	F	A	5/30/18		
6obe	B	A	4/1/20		
6obc	B	A	4/1/20		
6obo	C	A	4/1/20		X
6i6j	C	A	2/27/19		X
7mfu	B	A	6/2/21		X

7mfu	F	D	6/2/21		
7kgj	B	A	2/3/21		X
7kgk	B	A	2/3/21		
7now	C	D	4/7/21		X
7nqa	D	A	7/21/21		
6xzu	A	B	8/12/20		
7czd	A	B	7/14/21		

[1]Subset of cases used for benchmarking AlphaFold v.2.3.
[2]Subset of 100 cases used for benchmarking the use of bound input template structures.

References

1. Leman JK, Weitzner BD, Lewis SM, et al. Macromolecular modeling and design in Rosetta: recent methods and frameworks. *Nat Methods.* 2020;17(7):665-680.
2. Alford RF, Leaver-Fay A, Jeliazkov JR, et al. The Rosetta All-Atom Energy Function for Macromolecular Modeling and Design. *J Chem Theory Comput.* 2017;13(6):3031-3048.
3. Conway P, Tyka MD, DiMaio F, Konerding DE, Baker D. Relaxation of backbone bond geometry improves protein energy landscape modeling. *Protein Sci.* 2014;23(1):47-55.
4. Pierce BG, Hourai Y, Weng Z. Accelerating protein docking in ZDOCK using an advanced 3D convolution library. *PLoS One.* 2011;6(9):e24657.
5. Vreven T, Hwang H, Weng Z. Integrating atom-based and residue-based scoring functions for protein-protein docking. *Protein Sci.* 2011;20(9):1576-1586.
6. Basu S, Wallner B. DockQ: A Quality Measure for Protein-Protein Docking Models. *PLoS One.* 2016;11(8):e0161879.
7. Brenke R, Hall DR, Chuang GY, et al. Application of asymmetric statistical potentials to antibody-protein docking. *Bioinformatics.* 2012;28(20):2608-2614.
8. Zhang Y, Skolnick J. Scoring function for automated assessment of protein structure template quality. *Proteins.* 2004;57(4):702-710.
9. Fu L, Niu B, Zhu Z, Wu S, Li W. CD-HIT: accelerated for clustering the next-generation sequencing data. *Bioinformatics.* 2012;28(23):3150-3152.
10. Jumper J, Evans R, Pritzel A, et al. Highly accurate protein structure prediction with AlphaFold. *Nature.* 2021;596(7873):583-589.
11. *'NACCESS'* [computer program]. Department of Biochemistry and Molecular Biology, University College, London.1993.
12. Wallner B. AFsample: improving multimer prediction with AlphaFold using massive sampling. *Bioinformatics.* 2023;39(9).
13. Camacho C, Coulouris G, Avagyan V, et al. BLAST+: architecture and applications. *BMC bioinformatics.* 2009;10:421.
14. Dunbar J, Krawczyk K, Leem J, et al. SAbDab: the structural antibody database. *Nucleic Acids Res.* 2014;42(Database issue):D1140-1146.
15. Guest JD, Vreven T, Zhou J, et al. An expanded benchmark for antibody-antigen docking and affinity prediction reveals insights into antibody recognition determinants. *Structure.* 2021.
16. Yin R, Feng BY, Varshney A, Pierce BG. Benchmarking AlphaFold for protein complex modeling reveals accuracy determinants. *Protein Sci.* 2022;31(8):e4379.
17. Xu J, Zhang Y. How significant is a protein structure similarity with TM-score = 0.5? *Bioinformatics.* 2010;26(7):889-895.
